# Molecular Engineering of Interlayer Exciton Delocalization in 2D Perovskites

**DOI:** 10.1021/jacs.5c05621

**Published:** 2025-08-21

**Authors:** Yorrick Boeije, Fabian Lie, Miloš Dubajić, Ediz Garip, Arthur Maufort, Raisa-Iona Biega, Stijn Lenaers, Mylène Sauty, Pratyush Ghosh, Aleksandar Radić, Amélie Loher, Paola La Magna, Hayden Salway, Arjun Ashoka, Xian Wei Chua, Qichun Gu, Kristof Van Hecke, Laurence Lutsen, Dirk Vanderzande, Akshay Rao, Wouter T. M. Van Gompel, Linn Leppert, Samuel D. Stranks

**Affiliations:** † Department of Chemical Engineering and Biotechnology, 2152 University of Cambridge, Cambridge CB3 0AS, U.K.; ‡ Department of Physics, Cavendish Laboratory, University of Cambridge, Cambridge CB3 0HE, U.K.; § MESA+ Institute for Nanotechnology, 3230 University of Twente, Enschede 7522 NH, The Netherlands; ∥ 54496 Hasselt University, Institute for Materials Research (imo-imomec), Hybrid Materials Design (HyMaD), Martelarenlaan 42, B-3500 Hasselt, Belgium; ⊥ Department of Pure Mathematics and Mathematical Statistics, University of Cambridge, Cambridge CB3 0WB, U.K.; # Energyville, imo-imomec, Thor Park 8320, B-3600 Genk, Belgium; ∇ XStruct, Department of Chemistry, 98721 Ghent University, Krijgslaan 281-S3, B-9000 Ghent, Belgium

## Abstract

In recent years, significant progress has been made in improving the stability, photocurrent efficiency and charge transport properties of 2D hybrid perovskites, making them increasingly relevant for optoelectronic devices. Although these layered systems are typically considered quantum wells due to carrier confinement, an emerging strategy is to generate new perovskite functionalities with π-conjugated electroactive cores as spacer molecules, which introduce electronic coupling between the inorganic metal-halide and organic sublattices. Realizing these functionalities requires an understanding of how this coupling is achieved and how it affects exciton behavior. Using first-principles modeling and single-crystal optical spectroscopy, we find that the linker length (C_*x*_, where *x* = 2 or 4) controls the inorganic–organic electronic coupling and, therefore, the exciton properties of pyrene-alkylammonium (Pyr-C_*x*_)-based electroactive 2D perovskites. Whereas both (Pyr-C_2_)_2_PbI_4_ and (Pyr-C_4_)_2_PbI_4_ incorporate the π-conjugated core, only the latter has electroactive characteristics, as the longer linker length (*x* = 4) allows favorable π–π stacking that, together with energy alignment of organic and inorganic orbitals, results in interlayer organic–inorganic hybridization. This tailored hybrid coupling induces substantial exciton “leakage” through multiple PbI_4_^2–^ layers, enabling efficient interlayer exciton transport. By contrast, due to a type-I band alignment and orthogonal orientation of the π-systems with respect to the PbI_4_^2–^ layers in (Pyr-C_2_)_2_PbI_4_, the interlayer hybridization is lost, resulting in traditional quantum well properties. This study reveals new molecular engineering design principles to control excitons in 2D perovskites, emphasizing the importance of active π-core orientation and energetic band alignment—marking a critical step toward harnessing active organic cations in perovskite optoelectronics.

## Graphical abstract

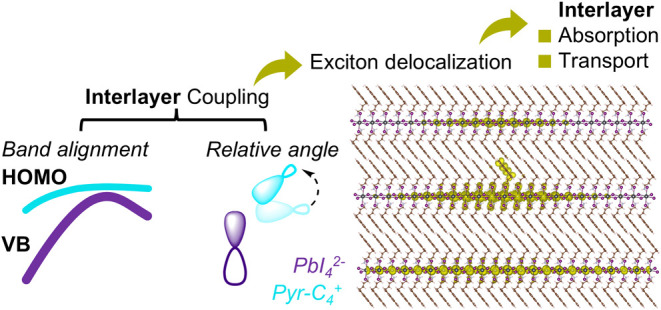

## Introduction

The rapid expansion in the diversity of 2D perovskites in the past few years has made them attractive candidates for multiple optoelectronic applications. These materials have the general formula R_2_A_*n*–1_M*_n_* X_3*n*+1_, where R is a large organic spacer cation, A is small monovalent cation, M is a divalent metal cation, X a monovalent halide anion, and n is the number of inorganic metal halide layers MX_4_^2–^ between the organic spacer layers. The organic spacer cation typically contains an alkyl ammonium chain that is connected to the inorganic layer via hydrogen bonding and electrostatic interactions. Although many previously used spacers are electronically and optically inert, a rapidly emerging area of research explores functionalized 2D perovskites, where the *active* organic spacer contains a π-conjugated core.^1,2^ Whereas spintronic functionalities have recently been enabled by chiral spacers,^3^ electroactive spacers provide a unique platform—beyond metal and halide substitution—to directly tune the bandgap, charge transport, and PL bandwidth. Introducing bulky π-conjugated organic spacers in either 2D perovskites (*n* = 1), quasi-2D perovskites (*n* > 2), or as passivating agents in 3D perovskites (*n* → ∞) has resulted in dramatically improved moisture and thermal stabilities,^4–7^ high photocurrent and power conversion efficiences,^8–11^ as well as boosted charge transport.^12,13^ Despite these promising achievements, the community’s current understanding of how π-conjugated organic spacers affect the electronic structure and carrier dynamics of 2D perovskites is limited, yet critical for tuning their photophysical properties and hence their use in applications such as photovoltaics, light-emitting diodes and X-ray scintillator devices.

Depending on the relative energies of the band edge states derived from the MX_4_^2–^ inorganic layer and the frontier orbitals of the R^+^ molecule, one can achieve a type-I or type-II band alignment. The band type alignment indicates the relative energy level ordering between the inorganic and organic sublattices and, therefore, controls where photoexcited carriers will localize.^14^ For most 2D perovskites, an electronically inactive spacer molecule is used, which is associated with a type-I band alignment, and therefore, 2D confined exciton dynamics dominated by the MX_4_^2–^ edge states.^15,16^ Alternatively, for sufficiently shallow highest occupied molecular orbitals (HOMOs) and deep lowest unoccupied molecular orbitals (LUMOs), energy transfer to the organic layer and subsequent singlet or triplet emission may occur.^17–21^ The type-II band alignment case favors photoinduced charge transfer and is typically associated with a quenched emission due to the spatial separation of photoexcited electrons and holes.^22–25^

These band alignment types are well-defined in the limit of negligible coupling between the organic and inorganic sublattices. However, strong interlayer orbital interactions (i.e., hybridization) can lead to out-of-plane (OOP) dispersion^13,26–29^ and the formation of charge transfer (CT) excitons,^30,31^ significantly complicating this picture. It is these situations we aim to understand in this paper, as strong OOP coupling boosts charge transfer^23,30,32^ and transport through the organic layers,^26,33–38^ which is relevant for various device types.

To computationally model inorganic–organic hybrid excitons in electroactive 2D perovskites, the electron–hole interaction should be taken into account explicitly, for example by using the Bethe-Salpeter Equation (BSE) of many-body perturbation theory. Although this framework has been applied for 2D perovskites containing inert organic spacers,^39–44^ to the best of our knowledge only one report exists for a 2D perovskite with an electroactive organic spacer.^45^ The 2D-layered perovskites containing a pyrene-alkylammonium cation (Pyr-C_*x*_) as the organic spacer (R), specifically (Pyr-C_2_)_2_PbI_4_ and (Pyr-C_4_)_2_PbI_4_, make a suitable set of representative model systems choice due to their available crystal structures (*x* = 2 ^8^ or *x* = 4 ^46^). Furthermore, the possibility of growing crystals enables the study of anisotropic optical properties and rules out morphological effects to explain differences in excitonic behavior. Previous work from Huang and co-workers has already demonstrated coupling between the inorganic layer and pyrene in polycrystalline thin films of (Pyr-C_4_)_2_PbI_4_ through the observation of triplet energy transfer.^21^ The computational study of Brédas and co-workers has predicted differences in band alignment for (Pyr-C_4_)_2_PbI_4_ and (Pyr-C_2_)_2_PbI_4_,^31^ but the significance for its optical properties has not been explored yet.

Here, we study the excitonic properties of (Pyr-C_2_)_2_PbI_4_ and (Pyr-C_4_)_2_PbI_4_ by combining first-principles many-body perturbation theory using the *GW*+BSE approach and single crystal optical spectroscopy and microscopy. We compare these samples to the literature reference (PEA)_2_PbI_4_, a 2D perovskite containing an electronically benign spacer. We observe substantial differences in exciton behavior for (Pyr-C_4_)_2_PbI_4_ and (Pyr-C_2_)_2_PbI_4_, despite the structural similarity of the organic spacer. The length of the alkyl chain linking the active π-core and 2D PbI_4_^2–^ sheet emerges as a key parameter to control band alignment, interlayer exciton hybridization and, therefore, exciton dynamics and transport. Whereas typical quantum-well-like behavior is observed in (Pyr-C_2_)_2_PbI_4_ and (PEA)_2_PbI_4_, including strong and sharp excitonic emission, weak out-of-plane absorption, and predominant lateral transport, (Pyr-C_4_)_2_PbI_4_ has a quenched emission with moderate out-of-plane absorption and significant vertical transport. The longer linker length unlocks a more favorable π–π stacking, which results in stronger interlayer hybridization through inward tilting of the pyrene π-core, rationalizing the improved electronic coupling between organic and inorganic sublattices despite the longer interlayer distance.

## Results

### Linker-Length-Induced Tuning of Inorganic–Organic Band Alignment and Hybridization

Thin single crystals of (PEA)_2_PbI_4_, (Pyr-C_2_)_2_PbI_4_ and (Pyr-C_4_)_2_PbI_4_ were grown on glass coverslips using the antisolvent vapor-assisted capping crystallization (AVCC) approach as detailed in the Methods section.^47^ X-ray diffraction patterns show preferential horizontal growth with equidistant reflections characteristic of 2D perovskites. We derived interlayer distances of 16.3, 24.6, and 25.4 Å for (PEA)_2_PbI_4_, (Pyr-C_2_)_2_PbI_4_, and (Pyr-C_4_)_2_PbI_4_, respectively, which are in close agreement with the interlayer distances derived from single crystal XRD data in earlier published works (Figure S3).^8,46,48^ Their crystal structures are plotted in Figure 1a–c with derived structure parameters provided in Supplementary Table 1. The in-plane (IP) and out-of-plane (OOP) directions are labeled to discuss anisotropic electronic and excitonic properties later in the text. The reflection spectra of representative flakes (Figure 1d, thickness ∼ 1 μm) are shown in Figure 1e, revealing the characteristic excitonic edge observed in 2D perovskites.^49^ The photoluminescence (PL) spectra of (PEA)_2_PbI_4_ and (Pyr-C_2_)_2_PbI_4_ (Figure 1f) show a sharp and strong emission that is spectrally close to their excitonic absorption edges as determined from reflection (Figure 1e) and transmission (Figure S4) spectra. The 2D confined nature of the PbI_4_^2–^ localized excitons gives rise to this strong emission with a quantum yield limited by trap densities.^50^ Strikingly, a much broader and weaker emission is observed in (Pyr-C_4_)_2_PbI_4_, indicating different recombination pathways of the photoexcited carriers. While variations in crystal thickness could, in principle, affect the shape of the PL spectrum through reabsorption, the distinct broad and red PL shoulder at ∼1.97–2.25 eV (550 nm–630 nm) observed only for (Pyr-C_4_)_2_PbI_4_, also appears in polycrystalline thin films—consistent with previous reports in which it was attributed to triplet emission (*vide infra*).^20,21^ Furthermore, reabsorption effects are expected to be saturated when the crystal thicknesses exceed the vertical diffusion length by at least an order of magnitude.^51^ In the following, we aim to understand how a subtle structural change in the organic spacer can induce such a drastic change in excitonic and emission properties.

**Figure 1:**
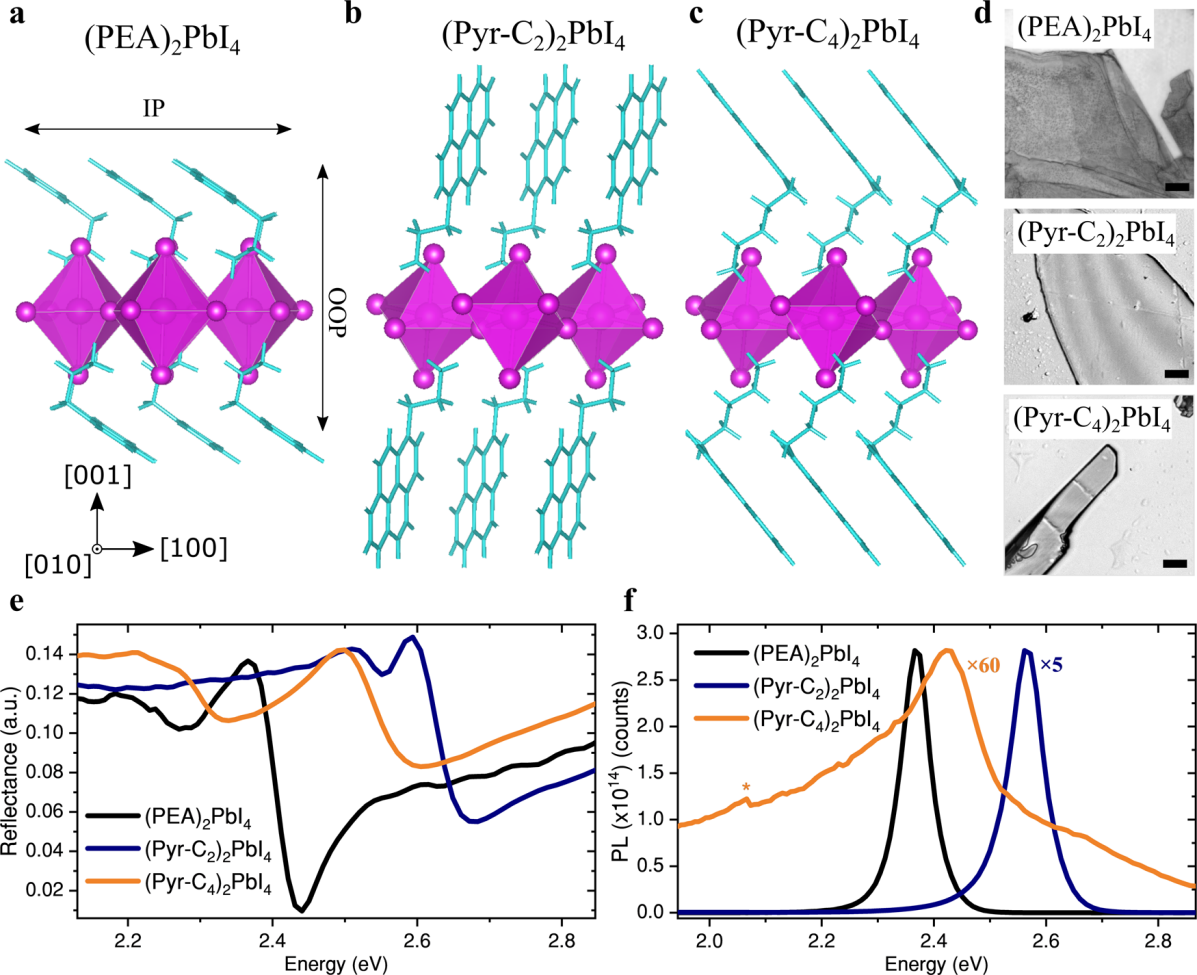
Electroactive pyrene core and linker length dictate optical properties. Crystal structures of (PEA)_2_PbI_4_ (a), (Pyr-C_2_)_2_PbI_4_ (b), and (Pyr-C_4_)_2_PbI_4_ (c), highlighting the organic and inorganic sublattices in light blue and magenta, respectively. Transmission images of unexfoliated single crystalline flakes obtained using the AVCC method (d). The scale bar indicates 10 μm for (Pyr-C_2_)_2_PbI_4_ and (Pyr-C_4_)_2_PbI_4_, and 100 μm for (PEA)_2_PbI_4_. Transmission spectra are provided in Supplementary Figure 5. Spatially averaged reflectance (e) and photoluminescence (PL) spectra (f) of crystalline flakes shown in (d). The excitation energy (wavelength) for the PL spectra was 3.06 eV (405 nm). The PL spectra of (Pyr-C_2_)_2_PbI_4_ and (Pyr-C_4_)_2_PbI_4_ were multiplied by 5 and 60, respectively, to normalize them to the PL maximum of (PEA)_2_PbI_4_. The asterisk marks the grating change at 2.07 eV (600 nm).

We computed the band structures of (PEA)_2_PbI_4_, (Pyr-C_2_)_2_PbI_4_, and (Pyr-C_4_)_2_PbI_4_ (Figure 2a–c, for complete band structures, see Figure S5) using a “one-shot” *G*_*0*_ *W*_*0*_ approach in which the zeroth-order Green’s function *G*_*0*_ and screened Coulomb interaction *W*_*0*_ are constructed from Density Functional Theory (DFT) eigenvalues and eigenfunctions using the PBE functional including spin–orbit coupling (SOC) self-consistently (*G*_*0*_ *W*_*0*_@PBE+SOC, see Methods). All structures exhibit a direct band gap with systematically underestimated absolute band gap energies *E*_g_ (Table 1) as usually encountered at this level of theory.^52^ Also consistent with the literature is the dominant contribution of Pb- and I-derived orbitals (“inorganic” in Figure 2) to the valence band maximum (VBM) and conduction band minimum (CBM) of (PEA)_2_PbI_4_ (Figure S5) as well as the strong IP dispersion (along the PbI_4_^2–^ layers, Γ → Y) and weak OOP dispersion (perpendicular to the PbI_4_^2–^ layers, Γ → Z). The band structure of (Pyr-C_2_)_2_PbI_4_ reveals a type-I band alignment similar to (PEA)_2_PbI_4_, although with a larger *E*_g_, which could be induced by significant octahedral distortion (Table S1 and Figure S7) or the larger interlayer distance.

**Figure 2:**
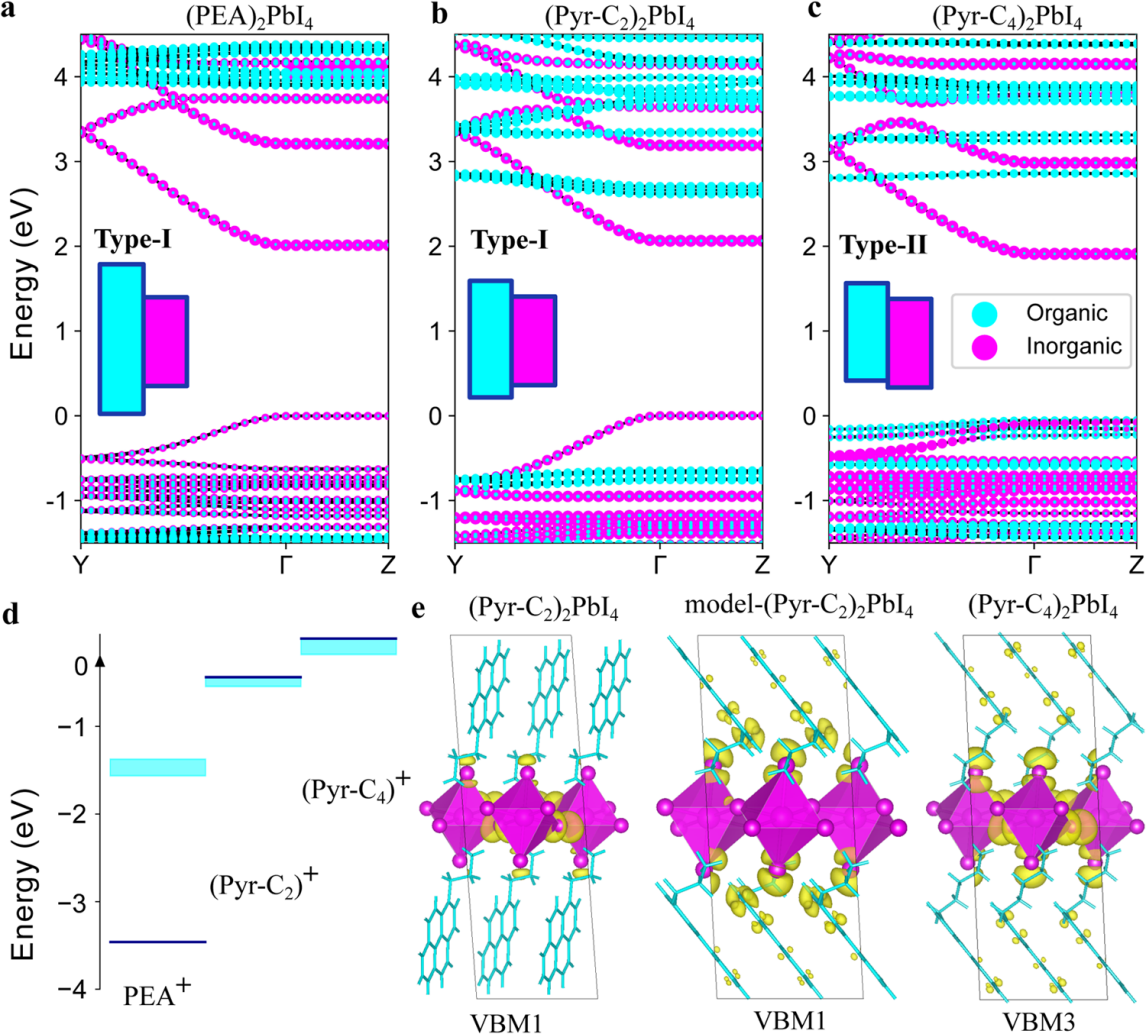
Band alignment and π–π stacking control interlayer hybridization. Band structures for (PEA)_2_PbI_4_ (a), (Pyr-C_2_)_2_PbI_4_ (b), and (Pyr-C_4_)_2_PbI_4_ (c) are calculated using DFT-PBE+SOC. A rigid shift is applied to the conduction bands to match the *G*_0_ *W*_0_@PBE+SOC band gap. GW corrections do not lead to a reordering of the energies of the inorganic valence band and the highest occupied organic orbitals, as shown in Figure S9. The band structures are aligned to the Pb 5s levels. In **k**-space the OOP direction corresponds to Γ → Z which exhibits negligible dispersion in all structures, irrespective of the inorganic/organic nature of the band. HOMO energy levels for isolated (gas-phase) charged spacer molecules (d). The energy of (Pyr-C_4_)^+^ is set to 0. The energy dispersion of the HOMO levels of the organic bands around Γ are displayed in cyan. Orbital charge densities of the highest occupied orbitals (Pyr-C_2_)_2_PbI_4_ (VBM1), and highest occupied hybridized orbitals in model-(Pyr-C_2_)_2_PbI_4_ (VBM1) and (Pyr-C_4_)_2_PbI_4_ (VBM3) (e).

**Table 1:** *G_0_W_0_* and BSE Band Structure and Absorption Results^a^

	*E*_g_ (eV)	μ_e,*x*/*y*_	μ_h,*x*/*y*_	*E*_b,IP/OOP_ (meV)	*E*_tr,IP/OOP_
(PEA)_2_PbI_4_	2.01	0.157/0.185	–0.213/–0.222	261/246	1.75/1.76
(Pyr-C_2_)_2_PbI_4_	2.06	0.176/0.168	–0.213/–0.222	251/242	1.81/1.82
(Pyr-C_4_)_2_PbI_4_	1.98	0.201/0.195	–1.202/–15.131	159/115	1.82/1.86

a *G*_0_ *W*_0_@PBE+SOC computed fundamental gap (*E*_g_) and electron (μ_e_) and hole (μ_h_) effective masses for both *x* and *y* directions. BSE computed exciton binding energies (*E*_b_) and transition energies (*E*_tr_) for the largest IP excitation along the [100] direction and the largest OOP excitation along the [001] direction. The corresponding IP (OOP) states for (PEA)_2_PbI_4_, (Pyr-C_2_)_2_PbI_4_, and (Pyr-C_4_)_2_PbI_4_ are 2 (5), 2 (4), and 10 (16), respectively.

By contrast, the band structure of (Pyr-C_4_)_2_PbI_4_ reveals a type-II band alignment with the valence band being dominated by organic-localized orbitals (for conceptual purposes referred to as HOMO) and the CBM being composed of inorganic orbitals. The change in band type alignment from (Pyr-C_2_)_2_PbI_4_ to (Pyr-C_4_)_2_PbI_4_ is at first sight counterintuitive considering that both 2D perovskites contain the same pyrene core, but is explained by a concurrent increase of the HOMO energy of the pyrene derivative (∼0.4 eV) and a drop of the VBM (∼0.1 eV) induced by the different alkyl chain lengths. The latter is due to a reduced octahedral tilting (Table S1), which also leads to a drop in the CBM (∼0.3 eV) and, therefore, a smaller inorganic band gap. It is well-known that the length and size of the organic spacer may result in bandgap shifts due to distinct steric interactions, as well as hydrogen bonding and electrostatic interactions with the inorganic layer, and is, therefore, not unique to electroactive spacers.^53^ What is unique here is that the subtle structural and electronic differences between (Pyr-C_2_)_2_PbI_4_ and (Pyr-C_4_)_2_PbI_4_ lead to a change in band alignment. As is apparent from Figure 2d, the HOMO shift is similar in the isolated Pyr-C_2_^+^ and Pyr-C_4_^+^ molecules, suggesting that the energy of the molecular electronic levels dominates this shift. Importantly, this shift is not the same in the neutral molecules, revealing that the inductive character of the ammonium group induces this HOMO shift that is preserved in the perovskite lattice (Table S2).

In addition to changing the band alignment, the differences in linker length also result in a different degree of inorganic–organic interlayer hybridization. The interlayer coupling is dependent on the energetic proximity of the VBM and HOMO, as well as the relative orientation of those orbitals. Due to the enhanced flexibility of the longer alkyl chain in (Pyr-C_4_)_2_PbI_4_, neighboring (filled) π-orbitals of the pyrene cores interact much more strongly, giving rise to H-aggregate like (face-to-face) π–π stacking (Figure 2e, see Figure S11 for more orbital density plots).^54^ This favorable H-aggregation results in an inward tilting of the π-orbitals, allowing hybridization with the valence bands consisting of iodide p-orbitals. Hence, the interlayer hybridization at the VBM is achieved by a correct energy alignment and structural arrangement induced by the tilting angle of pyrene with respect to PbI_4_^2–^. The H-aggregation also results in a strongly reduced hole effective mass along the π–π stacking direction ([100]) compared to its orthogonal direction ([010], Table 1), which is apparent from differences in Γ → Y and Γ → B dispersion (Figure S5). This anisotropy of the hole effective mass is absent in (Pyr-C_2_)_2_PbI_4_ and (PEA)_2_PbI_4_.

The absence of interlayer hybridization in (Pyr-C_2_)_2_PbI_4_ is explained by the pyrene π-orbitals that are orthogonal to the valence band and by the larger energetic HOMO-VBM separation. To verify the importance of the inward tilting of the pyrene π-core to achieve hybridization, we built a model-(Pyr-C_2_)_2_PbI_4_ system with a forced inward tilting to mimic the structural arrangement in (Pyr-C_4_)_2_PbI_4_ (Figure 2e and Figure S12 for band structure). Despite the initially less favorable HOMO-VBM energy gap, the hypothetical model-(Pyr-C_2_)_2_PbI_4_ has an even larger interlayer hybridization of the VB than (Pyr-C_4_)_2_PbI_4_, which is likely the result of the closer proximity between the pyrene π-orbitals and iodide p-orbitals. Note that whereas the VB hybridization is substantial, the CB hybridization is much weaker due to the Pb-dominated nature of these bands.

We emphasize that predicting the band alignment type in (Pyr-C_2_)_2_PbI_4_ and (Pyr-C_4_)_2_PbI_4_ based on the simplistic consideration of frontier orbital energy levels of isolated pyrene derivatives^55–58^ and the VBM of a typical 2D perovskite with an inert organic cation such as (PEA)_2_PbI_4_ ^59^ would not provide the same conclusions due to the subtle linker length induced orbital shifts discussed above. Similarly, since fine structural details control the hybridization, we emphasize the importance of performing electronic structure calculations on an experimentally determined geometry. Although the type of band alignments for (Pyr-C_4_)_2_PbI_4_ and (Pyr-C_2_)_2_PbI_4_ that we obtain are in line with calculations from Brédas and co-workers,^31^ the inorganic–organic band offsets are different, which we attribute to differences in the molecular stacking in the computational model of (Pyr-C_2_)_2_PbI_4_ used (an H-aggregate like π–π stacking similar to that of (Pyr-C_4_)_2_PbI_4_ was assumed by Brédas and co-workers for (Pyr-C_2_)_2_PbI_4_, which differs from the crystal structure that was determined experimentally) and the use of a different level of theory.

The striking band alignment difference between (Pyr-C_4_)_2_PbI_4_ and (Pyr-C_2_)_2_PbI_4_ is consistent with the much weaker and broader PL in (Pyr-C_4_)_2_PbI_4_ compared to the PL observed in (Pyr-C_2_)_2_PbI_4_, which is strong and sharp, qualitatively similar to the emission from (PEA)_2_PbI_4_, due to the 2D confined nature of the PbI_4_^2–^ localized excitons (Figure 1f). Quenching of the PL is indicative of a type-II band alignment where an energetic gradient drives *interlayer* charge separation,^60–62^ as opposed to recent *intralayer* exciton dissociation mechanisms in type-I 2D perovskites.^63–67^ The broad red PL shoulder at ∼1.97–2.25 eV (550 nm–630 nm) in the spectrum of (Pyr-C_4_)_2_PbI_4_ has been assigned to phosphorescence from pyrene due to triplet­(-excimer) states formed through triplet energy transfer from the inorganic framework,^20,21^ consistent with its photoluminescence excitation (PLE) spectrum (Figure S13) and its longer lifetime compared to the PbI_4_^2–^ exciton (Figure S14). If we assume that a CT-mediated two-step energy transfer mechanism is active for (Pyr-C_4_)_2_PbI_4_,^17,68–70^ the absence of any red PL for (Pyr-C_2_)_2_PbI_4_ can be explained by CT being energetically unfeasible in this material, turning off such an efficient energy transfer pathway. As a result, fast radiative decay of the bound exciton kinetically outcompetes the much slower triplet energy transfer, despite the latter being thermodynamically favorable. The similar shape of the PLE and absorbance spectra for (Pyr-C_2_)_2_PbI_4_ is consistent with its type-I band alignment and lack of any CT-induced energy transfer (Figure S13). Finally, the incomplete quenching of the excitonic emission in (Pyr-C_4_)_2_PbI_4_ at ∼512 nm is explained by the small HOMO-VBM offset of ∼10–20 meV (Figure 2c), which is close to room temperature thermal energy (∼25 meV) and phonon energies of the inorganic framework,^71,72^ leading to a significant population of PbI_4_^2–^ localized excitons due to dynamic disorder and (interlayer) exciton–phonon coupling.^32,73–75^

### Interlayer Exciton Delocalization

The combination of an electroactive π-core and spacer linker length allows us to tune the electronic coupling between the inorganic and organic layers and, thereby, the band alignment between the two layers. Here, we aim to understand how this tunable electronic coupling affects the excitonic properties of the 2D perovskite. We measured linear polarization-resolved reflection spectra for bulky crystals (see Methods) of (PEA)_2_PbI_4_ and (Pyr-C_4_)_2_PbI_4_ along both crystal planes (Figure 3a). Optical images and crystal thicknesses are provided in Figure S15. We computed the degree of anisotropy (Δ*R*/*R*, see Methods for details) by measuring the relative difference of the reflectance spectra for two orthogonal polarizations (see Figure S16 for the individual reflectance spectra). Despite significant efforts, we were unable to grow sufficiently large bulk crystals of (Pyr-C_2_)_2_PbI_4_ to be able to optically access the OOP axis. The excitonic reflection is completely isotropic along crystal plane 1 for (PEA)_2_PbI_4_, yet highly anisotropic along crystal plane 2, which includes the OOP axis. The non-negligible OOP reflection component for excitons in (PEA)_2_PbI_4_ (Figure S16) is consistent with previous polarization-resolved optical measurements on single crystal 2D perovskites,^49,76–78^ magneto-optical measurements^79,80^ as well as theoretical modeling.^41–43,81^ On the contrary, (Pyr-C_4_)_2_PbI_4_ exhibits a markedly reduced reflection anisotropy—approximately six times smaller—indicating a relative enhancement in the OOP absorption coefficient. We note that the reflection anisotropy is associated with birefringence, which results in reflectance intensity differences below the absorption onset (Figure S16).^49^

**Figure 3:**
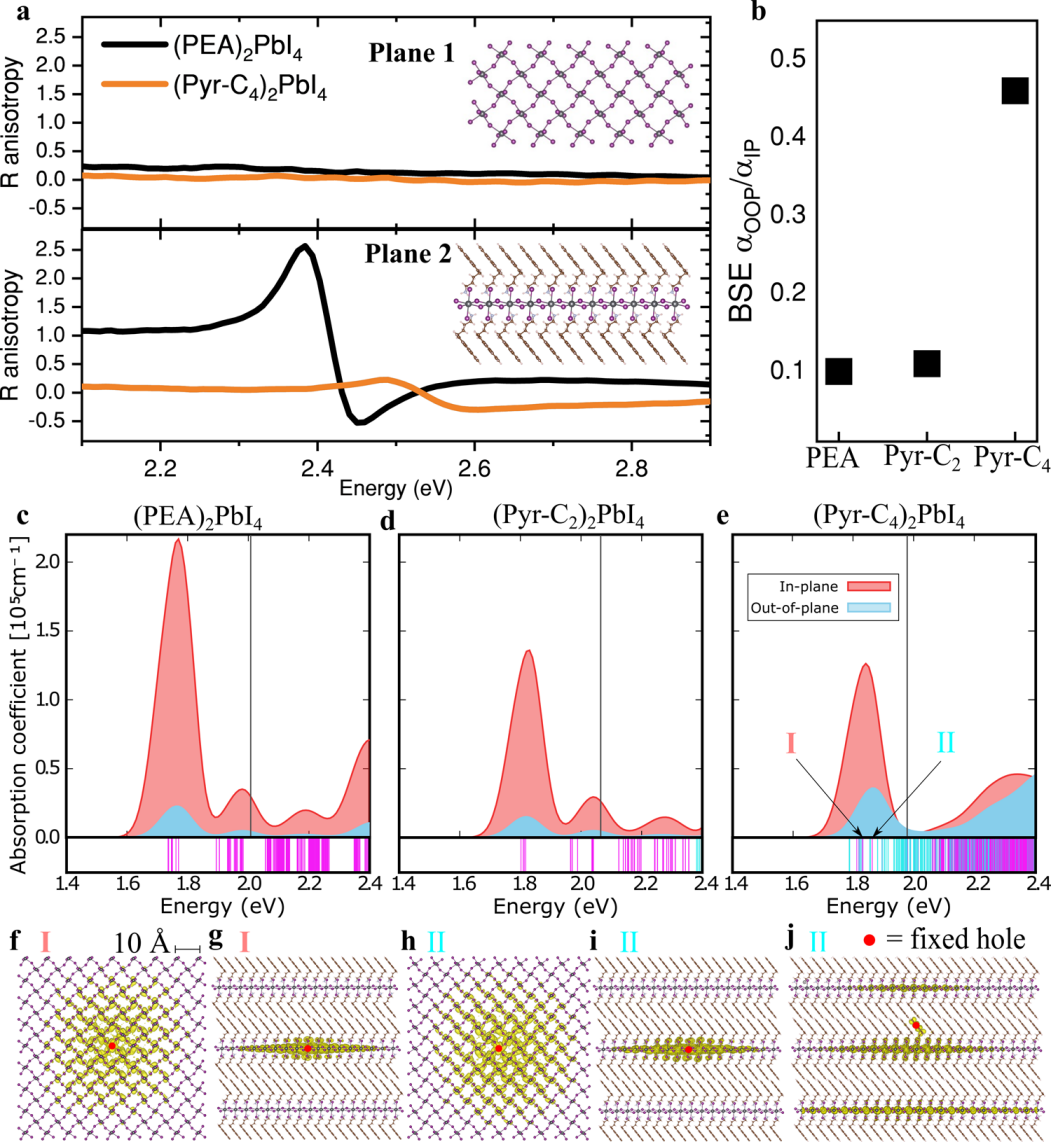
Charge transfer-induced interlayer exciton delocalization enhances out-of-plane absorption strength. Optical polarization reflectance anisotropy spectra for both crystal planes in bulk crystals of (PEA)_2_PbI_4_ and (Pyr-C_4_)_2_PbI_4_ (a). *R* anisotropy is measured as (*R_a_*-*R_b_*)/*R_a_*, where *a* and *b* are crystallographic axes that define a plane. These planes are visualized for (Pyr-C_4_)_2_PbI_4_, where Plane 1 contains both in-plane (IP) axes and Plane 2 contains one IP axis and the OOP axis. *G*_0_ *W*_0_+BSE calculated ratios of OOP and IP absorption coefficients (α_OOP_/α_IP_) (b). These ratios were acquired by integrating over all transitions of the main excitonic peak in the *G*_0_ *W*_0_+BSE absorption spectra for (PEA)_2_PbI_4_ (c), (Pyr-C_2_)_2_PbI_4_ (d), and (Pyr-C_4_)_2_PbI_4_ (e). The black vertical line indicates the *G*_0_ *W*_0_@PBE+SOC band gap. Light-blue (red) component indicates the OOP (IP) absorption. The bars below the spectra indicate excitation energies, which are colored either magenta or cyan depending on the degree of inorganic–organic character. The brightest IP and OOP excitons are indicated with I and II, respectively. Exciton wave function density of exciton I (f, g) and II for (Pyr-C_4_)_2_PbI_4_ with the hole fixed on a lead atom (h, i), and on the pyrene unit (j).

The experimentally observed OOP absorption is surprisingly strong considering that, at the single-particle level of theory, the inorganic and organic bands in (Pyr-C_4_)_2_PbI_4_ are only weakly hybridized (Figure S11) despite their energetic proximity (Figure 2c). Furthermore, both inorganic and organic bands are rather flat along the OOP direction (Γ → Z), resulting in a large effective hole mass (Table 1). We therefore calculated the optical absorption spectra of (PEA)_2_PbI_4_, (Pyr-C_2_)_2_PbI_4_, and (Pyr-C_4_)_2_PbI_4_, explicitly including electron–hole interactions in the *G*_0_ *W*_0_+BSE approach (see Methods). The ratios of OOP and IP absorption coefficient of the main excitonic peaks (α_OOP_/α_IP_, Figure 3b), derived from the spectra (Figure 3c–e) show consistency with the experimental crystal axis-dependent reflection. The polarization dependence of the absorption spectrum of (PEA)_2_PbI_4_ is isotropic for both IP axes but has significant anisotropy with respect to the OOP axis, giving rise to a small α_OOP_/α_IP_. The α_OOP_/α_IP_ value increases by approximately 5-fold from (PEA)_2_PbI_4_ to (Pyr-C_4_)_2_PbI_4_, despite the significantly larger interlayer distance (respectively 16 and 25 Å, Table S1), which should in a first approximation decrease the OOP coupling.^41^ We can not attribute this increase purely to an enhanced dielectric constant associated with the larger π-conjugated pyrene core, as α_OOP_/α_IP_ is much smaller in (Pyr-C_2_)_2_PbI_4_ compared to (Pyr-C_4_)_2_PbI_4_. Furthermore, the increased octahedral distortion compared to (PEA)_2_PbI_4_ can also not explain the increased OOP absorption for (Pyr-C_4_)_2_PbI_4_, as (Pyr-C_2_)_2_PbI_4_ experiences even larger degrees of octahedral distortion than (Pyr-C_4_)_2_PbI_4_ (Table S1).

Consequently, we argue that the close energetic proximity of the pyrene HOMO to the PbI_4_^2–^ VBs in (Pyr-C_4_)_2_PbI_4_ gives rise to strong organic–inorganic orbital mixing and, therefore, charge transfer (CT) character in the lower-energy excitonic transitions,^45^ resulting in a stronger OOP coupling with multiple PbI_4_^2–^ layers and therefore a boost in the OOP absorption coefficient. The exciton densities for the brightest IP and OOP excitons in (Pyr-C_4_)_2_PbI_4_ (Figure 3f–j) support this mechanism. Whereas the IP exciton is well described by nearly pure inorganic–inorganic transitions (Figure 3f and g), the OOP exciton is better described by a mixture of inorganic–inorganic transitions (Figure 3h and i) and hybrid inorganic–organic transitions (Figure 3j). In fact, the brightest OOP exciton contains ∼30% hole wave function localized on the pyrene unit, which is illustrated by the high density of cyan bars in Figure 3e. It is the large contribution of hybrid inorganic–organic transitions to the OOP exciton—and therefore the CT character—that provides sufficient OOP coupling to delocalize the electron wave function across ∼4 inorganic layers. This situation is illustrated in Figure 3j where the hole is localized on pyrene. On the contrary, for pure inorganic–inorganic transitions—i.e., when the hole is localized on lead as shown in Figure 3i—the electron wave function is localized to a single inorganic layer. The relationship between inorganic–organic CT character and OOP oscillator strength, along with a detailed analysis of the exciton fine structure, is further discussed in Supplementary Note 1. Interestingly, a similar enhancement of OOP oscillator strength was also predicted for the as-of-yet unsynthesized (Pyr-C_3_)_2_PbI_4_ compound using time-dependent DFT.^31^

On the other hand, due to the large energy gap between organic and inorganic orbitals in (Pyr-C_2_)_2_PbI_4_, the OOP coupling is much weaker, leading to only minor OOP absorption (Figure 3d). As a result, excitons in (Pyr-C_2_)_2_PbI_4_ behave more like those in a traditional quantum well 2D perovskite compared to (Pyr-C_4_)_2_PbI_4_, despite the presence of the π-conjugated pyrene core. This behavior is consistent with its type-I band alignment (Figure 2b) and bright excitonic photoluminescence (Figure 1f).^31^

### Exciton Delocalization Drives Interlayer Transport

Next, we investigate how the distinct nature of excitons in (PEA)_2_PbI_4_, (Pyr-C_2_)_2_PbI_4_, and (Pyr-C_4_)_2_PbI_4_ affects their transport behavior with interferometric femtosecond transient absorption microscopy (TAM). In this experiment (see Methods for further details and Figure 4a for visualization), a near-diffraction limited pump excites a local exciton carrier distribution that diffuses away from the central spot as a result of a carrier gradient.^82,83^ The resulting expansion is then imaged with a wide-field probe for varying pump–probe time delays and is generally fitted to temporally broadening Gaussian functions (i.e., an increasing width, σ) to retrieve the IP exciton diffusion constant. We apply a modified version of the analytical optical model developed by Ashoka et al. to be able to fit each interferometric Δ*T*/*T*(*x*,*y*,*t*) image and quantify both IP and OOP transport (Supplementary Note 2).^84^ Briefly, in this approach the spatial interference between the unperturbed and pump-induced perturbed probe waves is described as the near-field electric field Fresnel diffraction of a three-dimensional Gaussian beam attenuated through the crystal depth (*z*). Importantly, the spatial exciton distribution is expressed by the complete complex transient refractive index function Δ*ñ*, and thus prior knowledge is required about both the real (Δ*n*) and imaginary (Δ*k*) photoinduced complex refractive index, which we retrieve through a variational Kramers–Kronig analysis of static transmission and transient absorption data (Figure S17).^85^ Hence, by modeling Δ*T*/*T*(*x*,*y*,*t*) in this way, we account for (i) exciton decay through the real Δ*n* and imaginary Δ*k* part of the refractive index, (ii) IP diffusion through σ (lateral width) and (iii) OOP diffusion through Δ*z* (vertical gradient). The IP diffusion component characterizes lateral diffusion of excitons across the inorganic layers, and the OOP diffusion component characterizes vertical diffusion perpendicular to the inorganic layers, because the 2D perovskite crystalline flakes consist of horizontally grown layers (Figure S3).

**Figure 4:**
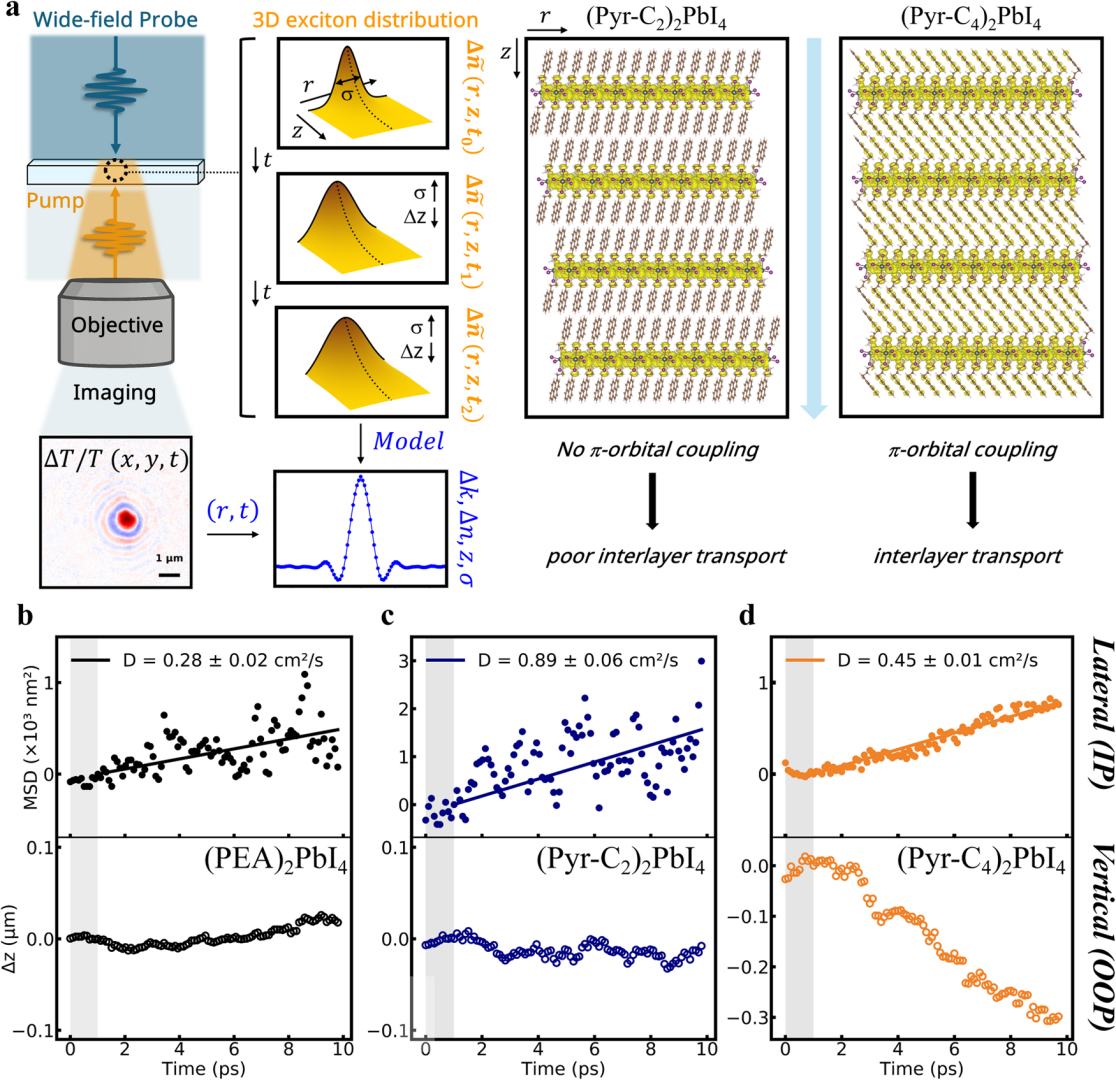
Transient absorption microscopy reveals varying degrees of in-plane (IP) and out-of-plane (OOP) diffusion. Schematic illustration of experiment (a). Focused pump excitation generates a three-dimensional exciton carrier distribution Δ*ñ*, which undergoes lateral and vertical diffusion as characterized by an increasing width (σ) and decreasing vertical gradient (Δz). The diffusion is measured using a counter-propagating wide-field probe. The transmitted probe wave diffraction pattern depends on the degree of interlayer transport, tuned by the choice of organic spacer. Fitted mean square displacement (MSD) and Δz for (PEA)_2_PbI_4_ (b), (Pyr-C_2_)_2_PbI_4_ (c), and (Pyr-C_4_)_2_PbI_4_ (d) single crystals. The MSD plots were fitted to a linear function to retrieve the IP diffusion constant. The other fitted parameters, Δ*n* and Δ*k*, are provided in Figure S19. The same pump energy (wavelength) of 2.76 eV (450 nm) was used, but varying probe wavelengths due to the different ground-state bleach spectral ranges in these materials. λ_probe_ = 530 nm (2.34 eV), 480 nm (2.58 eV), and 500 nm (2.48 eV) for (PEA)_2_PbI_4_, (Pyr-C_2_)_2_PbI_4_, and (Pyr-C_4_)_2_PbI_4_, respectively. We have excluded the 0–1 ps temporal region from the linear fit to reduce contributions from carrier cooling and exciton–exciton annihilation, as indicated with the gray box.

The picosecond decay kinetics of Δ*T*/*T* are provided in Figure S18, showing a slightly faster decay of (Pyr-C_2_)_2_PbI_4_ compared to (Pyr-C_4_)_2_PbI_4_. We retrieved σ^2^(*t*) and Δ*z*(*t*) for (PEA)_2_PbI_4_, (Pyr-C_2_)_2_PbI_4_, and (Pyr-C_4_)_2_PbI_4_ single crystalline flakes (Figure 4b–d), and extracted IP diffusion coefficients (*D*) in cm^2^/s by fitting the mean square displacement (MSD = σ^2^(*t*) – σ^2^(0)) to 2 × *D* × *t*. Applying this equation is justified by (i) the observed linear relation between MSD and *t*, disregarding a sub- or superdiffusive diffusion regime and (ii) the monomolecular decay kinetics observed in the low pump fluence regime employed (Figure S18).^86–88^ The sign of the fitted Δ*n* and Δ*k* parameters is consistent with our Kramers–Kronig analysis (Figure S17), and their temporal behavior represents the decay of excitons correctly (Figure S19). Representative Δ*T*/*T* images with their corresponding fits are provided in Supplementary Figure 20.

The extracted IP diffusion constants for (PEA)_2_PbI_4_, (Pyr-C_2_)_2_PbI_4_, and (Pyr-C_4_)_2_PbI_4_ (see Figure 4b–d, 0.28 ± 0.02, 0.89 ± 0.06 cm^2^/s and 0.45 ± 0.01 cm^2^/s, respectively) are roughly an order of magnitude larger than the diffusion constant measured with transient absorption microscopy of a butyl-ammonium 2D perovskite,^89^ but fall within the range of transient PL-derived diffusion constants for various 2D perovskites.^90–95^ Importantly, and consistent with our justifications presented above, diffusion constants for (Pyr-C_2_)_2_PbI_4_ and (Pyr-C_4_)_2_PbI_4_ are independent of pump fluence (Figure S21). The negligible Δ*z* temporal evolution for (PEA)_2_PbI_4_ and (Pyr-C_2_)_2_PbI_4_ (Figure 4b and c, bottom panels) is another manifestation of the PbI_4_^2–^ quantum well localized nature of its excitons without significant OOP coupling, enforced by the type-I band alignment. Strikingly, *z* reduces significantly in (Pyr-C_4_)_2_PbI_4_ (Figure 4d, bottom panel), indicating a decreasing exciton *z*-gradient distribution due to vertical diffusion. Although a recent report has revealed a ∼10^4^ smaller OOP exciton diffusion compared to IP diffusion in (PEA)_2_PbI_4_ thin films,^96^ other reports revealed that OOP exciton diffusion constants in typical 2D perovskites are only a few times smaller than IP diffusion constants,^95,97^ a much larger ratio compared to electron/hole transport,^98,99^ and can be explained by either FRET or coherent energy transfer mechanisms.^100^ The much larger Δ*z* evolution in (Pyr-C_4_)_2_PbI_4_ compared to (Pyr-C_2_)_2_PbI_4_ is inconsistent with a vertical FRET hopping mechanism, considering the larger interlayer distance (∼0.8 Å, Figure S3). Instead, we hypothesize that the stronger organic–inorganic interlayer coupling induced exciton delocalization in (Pyr-C_4_)_2_PbI_4_ drives coherent energy transfer across the layers.

Although the enhanced lattice rigidity enforced by the stronger π–π stacking in (Pyr-C_4_)_2_PbI_4_ compared to (Pyr-C_2_)_2_PbI_4_ is expected to reduce exciton–phonon coupling and thereby improve lateral transport,^90,94,101^ the observed differences in lateral diffusion likely arise from a combination of factors. In particular, the larger effective mass associated with the interlayer character of excitons in (Pyr-C_4_)_2_PbI_4_ may play a role (Table 1). Additionally, the IP diffusion in (Pyr-C_4_)_2_PbI_4_ is influenced by the strongly time-varying z-gradient (Supplementary Note 2).

## Discussion

We have shown how the combination of a π-conjugated molecule and spacer linker length can be used to tune excitonic properties in a 2D perovskite (Pyr-C_*x*_)_2_PbI_4_ (where *x* = 2 or 4). Excitons in (Pyr-C_2_)_2_PbI_4_ are confined in two dimensions like in a traditional quantum well perovskite, such as (PEA)_2_PbI_4_, giving rise to strong and sharp emission, and predominantly in-plane diffusion. Excitons in (Pyr-C_4_)_2_PbI_4_ have mixed inorganic–organic characteristics and therefore strong interlayer delocalization, resulting in “quasi-quantum well” behavior, which is associated with stronger out-of-plane absorption and transport.

These results highlight the potential for electroactive organics in perovskite photovoltaic devices, where strong inorganic–organic orbital coupling could enable improved transport properties. Promising tailored transport properties have already been reported for pyrene-based 2D lead iodide^57^ and double perovskites,^38^ 2D/3D heterostructures^102^ and surface-modified 3D perovskites.^8^ An increasingly rich literature on perovskite optoelectronics is emerging by employing a wide variety of electroactive molecular structures.^11,103^ The general motivations for using these molecules are enhanced material stability, facilitated charge separation, and improved transport. Our previous work on carbazole-based 2D perovskites (Cz-C_*x*_)_2_PbI_4_ (where *x* = 3, 4, or 5) showed that decreasing the spacer linker length resulted in stronger interlayer electronic coupling.^30^ Hence, the reduced interlayer coupling in (Pyr-C_2_)_2_PbI_4_ compared to (Pyr-C_4_)_2_PbI_4_ appears initially counterintuitive. This apparent contradiction is resolved in the present work by considering not only the distance between the π-conjugated core and PbI_4_^2–^ layer, but also their relative orientation. A longer linker length (*x* = 4) is required to achieve favorable intermolecular H-aggregate formation between the pyrene cores, allowing its π-orbitals to couple to the PbI_4_^2–^ valence band. The orthogonality between the π-orbitals and PbI_4_^2–^ valence bands rationalizes the lost interlayer electronic coupling in (Pyr-C_2_)_2_PbI_4_, emphasizing that a shorter linker length does not necessarily improve the coupling and, more importantly, that the mere presence of a π-conjugated molecule in the perovskite is not sufficient to gain electroactive characteristics. Whereas in (Cz-C_*x*_)_2_PbI_4_ fine-tuning of interlayer coupling is achieved with variations in linker length, a much stronger—and unexpectedly reversed—dependence is observed for (Pyr-C_*x*_)_2_PbI_4_. The new chemical design principles obtained in this work should spark the future design of inorganic–organic hybrid perovskite materials and interfaces where orientation, length and the nature of the organic spacer can be leveraged for realizing new properties.

We reveal how the inorganic–organic interlayer coupling in (Pyr-C_4_)_2_PbI_4_ enables significant interlayer exciton delocalization and consider its potential relevance for efficient vertical exciton transport mechanisms. Charge and exciton transport mechanisms in such a strongly hybridized system require further exploration, as it has a complex energetic landscape distinct from both their 2D and 3D perovskite analogues with electronically inert organic cations. Future work will focus on investigating how the exciton tuneability impacts carrier dynamics and transport mechanisms within optoelectronic devices in order to maximize the application potential of molecularly engineered 2D perovskites. In doing so, we aim to leverage the enhanced stability of 2D perovskites while mitigating the limitations associated with the typically insulating properties of organic spacer layers through optimized interactions between the electroactive organic spacer and inorganic layers. Moreover, the molecular-level understanding of interlayer electronic coupling developed in this work provides valuable design principles for optimizing interfacial transport across heterostructures in device stacks, such as at 2D/3D perovskite interfaces^102,104^ or perovskite/organic transport layer junctions.^105^ Achieving efficient charge and exciton transfer in such architectures, and thereby suppressing interfacial trapping and nonradiative voltage losses, requires not only careful band alignment but also precise control over molecular orientation—a challenge that can be addressed through the targeted molecular design strategies demonstrated in this work.

## Materials

### Chemicals and Reagents

All commercial chemicals and solvents were used without additional purification steps unless stated otherwise.

Lead­(II) iodide (99.99%) and γ-butyrolactone (GBL, >99%) were purchased from TCI Chemicals and stored in a nitrogen-filled glovebox to prevent water intrusion. HI (57 wt % in water, distilled, unstabilized), 2-phenylethylamine (99%), and tri-*n*-butyl phosphate (>99%) were purchased from Acros Organics. All other solvents were purchased from Fisher Scientific. HI was extracted with 9:1 chloroform/tri-*n*-butyl phosphate prior to use.

Details on the synthesis of the organic ammonium iodide salts can be found in the Supporting Information.

### Thin Film Deposition and Annealing

#### (Pyr-C_4_)_2_PbI_4_

Precursor solutions were prepared by dissolving PyrC_4_NH_3_I (0.08 M) and PbI_2_ (0.04 M) together in dry *N,N*-dimethylformamide (DMF) at 50 °C for 30 min under constant stirring. The resulting clear solutions were filtered through a syringe filter (0.2 μm pore size). Quartz substrates were cleaned by subsequent sonication in deionized water, acetone, and isopropanol (15 min each). Afterward, the substrates were treated with UV/ozone for 15 min. Film deposition was performed via spin coating 40 μL of the precursor solution at 4000 and 4000 rpm/s for 20 s. Thermal annealing was performed on a hot plate at 150 °C for 10 min. Film deposition and thermal annealing of the substrates were performed in a N_2_-filled glovebox. Prepared samples were kept in a glovebox and were only removed for analysis.

#### (Pyr-C_2_)_2_PbI_4_

Precursor solutions were prepared by dissolving PyrC_2_NH_3_I (0.075M) and PbI_2_ (0.0375 M) in a 9:1 mixture of dry *N,N*-dimethylformamide (DMF) and dimethyl sulfoxide (DMSO) at 50 °C for 2 h under constant stirring. The resulting clear solutions were filtered through a syringe filter (0.2 μm pore size). Quartz substrates were cleaned by subsequent sonication in deionized water, acetone, and isopropanol (15 min each). Afterward, the substrates were treated with UV/ozone for 15 min. Film deposition was performed via spin coating 40 μL of the precursor solution at 4000 and 4000 rpm/s for 20 s. Thermal annealing was performed on a hot plate at 100 °C for 10 min. Film deposition and thermal annealing of the substrates were performed in a N_2_-filled glovebox. Prepared samples were kept in a glovebox and were only removed for analysis.

#### (PEA)_2_PbI_4_

Precursor solutions were prepared by dissolving PEAI (0.08 M) and PbI_2_ (0.04 M) together in dry *N,N*-dimethylformamide (DMF) at 50 °C for 2 h under constant stirring. The resulting clear solutions were filtered through a syringe filter (0.2 μm pore size). Quartz substrates were cleaned by subsequent sonication in deionized water, acetone, and isopropanol (15 min each). Afterward, the substrates were treated with UV/ozone for 15 min. Film deposition was performed via spin coating 40 μL of the precursor solution at 4000 and 4000 rpm/s for 20 s. Thermal annealing was performed on a hot plate at 100 °C for 10 min. Film deposition and thermal annealing of the substrates were performed in a N_2_-filled glovebox. Prepared samples were kept in a glovebox and were only removed for analysis.

#### (PyrC_4_)­I

Precursor solutions were prepared by dissolving (PyrC_4_)I (0.1 M) in a 9:1 mixture of dry *N,N*-dimethylformamide (DMF) and dimethyl sulfoxide (DMSO) at 50 °C for 2 h under constant stirring. The resulting clear solutions were filtered through a syringe filter (0.2 μm pore size). Quartz substrates were cleaned by subsequent sonication in deionized water, acetone, and isopropanol (15 min each). Afterward, the substrates were treated with UV/ozone for 15 min. Film deposition was performed via spin coating 50 μL of the precursor solution at 2000 and 2000 rpm/s for 20 s. Film deposition was performed in a N_2_-filled glovebox. Prepared samples were kept in a glovebox and were only removed for analysis.

#### (PyrC_2_)­I

Precursor solutions were prepared by dissolving (PyrC_2_)I (0.1 M) in a 9:1 mixture of dry *N,N*-dimethylformamide (DMF) and dimethyl sulfoxide (DMSO) at 50 °C for 2 h under constant stirring. The resulting clear solutions were filtered through a syringe filter (0.2 μm pore size). Quartz substrates were cleaned by subsequent sonication in deionized water, acetone, and isopropanol (15 min each). Afterward, the substrates were treated with UV/ozone for 15 min. Film deposition was performed via spin coating 50 μL of the precursor solution at 2000 and 2000 rpm/s for 20 s. Film deposition was performed in a N_2_-filled glovebox. Prepared samples were kept in a glovebox and were only removed for analysis.

### Synthesis of Bulk Single Crystals

#### (Pyr-C_4_)_2_PbI_4_

The single crystals were grown using an antisolvent vapor-assisted crystallization approach^106^ in which the components are dissolved together in a good solvent (*y*-butyrolactone; GBL) and dichloromethane (DCM) antisolvent slowly diffuses into the GBL solution through the vapor phase. Specifically, Pyr-C_4_NH_3_I (0.2 M) and PbI_2_ (0.1 M) were dissolved together in dry GBL by stirring at 50 °C for 15 min. The precursor solution was filtered through a syringe filter (0.45 μm). The precursor solution (0.5 mL) was transferred to a small glass vial. The small vial (5 mL volume) was capped off with aluminum foil. A small hole was made in the aluminum foil. The small vial with the aluminum foil was put in a larger glass vial (20 mL volume). An amount of dry dichloromethane (1 mL) was injected in the gap between the two vials and the larger vial was capped off with a plastic cap and parafilm. The vials were left undisturbed at room temperature. After 2 days, small orange needle-like crystals were formed. At that moment, the lid of the larger vial was removed, an additional amount of dichloromethane (2 mL) was injected in the gap between the two vials, and the larger vial was again capped off with a plastic cap and parafilm. After another 5 days, larger orange needle-like crystals were harvested. The crystals were washed by depositing them in a glass vial filled with dry diethyl ether (4 mL), after which they were transferred to an empty glass vial. The crystals were subsequently dried under reduced pressure at room temperature.

#### (PEA)_2_PbI_4_

The single crystals were grown using an antisolvent vapor-assisted crystallization approach.^106^ Specifically, PEAI (2.0 M) and PbI_2_ (1 M) were dissolved together in dry GBL by stirring at 50 °C for 15 min. The precursor solution was filtered through a syringe filter (0.45 μm). The precursor solution (0.5 mL) was transferred to a small glass vial. The small vial (5 mL volume) was capped off with aluminum foil. A small hole was made in the aluminum foil. The small vial with the aluminum foil was put in a larger glass vial (20 mL volume). An amount of dry dichloromethane (1 mL) was injected in the gap between the two vials and the larger vial was capped off with a plastic cap and parafilm. The vials were left undisturbed at room temperature. After 3 days, orange needle-like crystals were harvested. The crystals were washed by depositing them in a glass vial filled with dry diethyl ether (4 mL), after which they were transferred to an empty glass vial. The crystals were subsequently dried under reduced pressure at room temperature.

### Synthesis of Thin Single Crystals on Glass Substrates

Single crystals on glass substrates were grown via the antisolvent vapor capping crystallization (AVCC) method reported by Lédée et al.^47^

#### (Pyr-C_4_)_2_PbI_4_

A precursor solution of PyrC_4_NH_3_I (0.91 M) and PbI_2_ (0.45 M) in GBL was stirred at 50 °C and filtered through a syringe filter (0.45 μm pore size). Four μL of this precursor was suspended on a glass substrate (22 × 22 mm) in a 100 mL vial. Subsequently, a second glass substrate was carefully placed on top (to sandwich the precursor between the two substrates), a GPC vial with 1.5 mL chloroform was placed on top, and the 100 mL vial was closed with a screw cap and parafilm. The setup was put in an incubator at 20 °C to prevent temperature fluctuations. The slow diffusion of chloroform vapor into the precursor leads to perovskite crystallization between the two glass slides. After 24 h, the substrates were taken out of the 100 mL vial, were carefully separated with a lancet, and were dipped for 1–2 s in a beaker with diethyl ether to remove precursor residues, leaving single-crystalline flakes attached to the glass surface.

#### (Pyr-C_2_)_2_PbI_4_

A precursor solution was prepared by dissolving Pyr-C_2_NH_3_I (0.45 M) and PbI_2_ (0.23 M) in a 3:1 mixture of dry γ-butyrolactone (GBL) and dry *N,N*-dimethylformamide (DMF) at 50 °C. 4 μL of this precursor was suspended on a glass substrate (22 × 22 mm) in a 100 mL vial. Subsequently, a second glass substrate was carefully placed on top (to sandwich the precursor between the two substrates), a GPC vial with 1 mL chloroform was placed on top, and the 100 mL vial was closed with a screw cap and parafilm. The setup was put in an incubator at 20 °C to prevent temperature fluctuations. The slow diffusion of chloroform vapor into the precursor leads to perovskite crystallization between the two glass slides. After 24 h, the substrates were taken out of the 100 mL vial, were carefully separated with a lancet, and were subsequently dipped for 1–2 s in a beaker with acetonitrile and a beaker with diethyl ether to remove precursor residues, leaving single-crystalline needles attached to the glass surface.

#### (PEA)_2_PbI_4_

A precursor solution of PEAI (2.0 M) and PbI_2_ (1 M) in GBL was stirred at 50 °C and filtered through a syringe filter (0.45 μm pore size). Four μL of this precursor was suspended on a glass substrate (22 × 22 mm) in a 100 mL vial. Subsequently, a second glass substrate was carefully placed on top (to sandwich the precursor between the two substrates), a GPC vial with 1.5 mL dichloromethane was placed on top, and the 100 mL vial was closed with a screw cap and parafilm. The setup was put in an incubator at 20 °C to prevent temperature fluctuations. The slow diffusion of chloroform vapor into the precursor leads to perovskite crystallization between the two glass slides. After 24 h, the substrates were taken out of the 100 mL vial, were carefully separated with a lancet, and were dipped for 1–2 s in a beaker with diethyl ether to remove precursor residues, leaving single-crystalline flakes attached to the glass surface.

## Methods

### First-Principles Calculations

Density Functional Theory (DFT) calculations are carried out using the Quantum Espresso software package^107,108^ using the exchange-correlation functional by Perdew, Burke, and Ernzerhof (PBE).^109^ We use a set of norm-conserving fully relativistic pseudopotentials (ONCVPSP v 3.3.0),^110–112^ with the following atomic configurations: 5d^10^6s^2^6p^2^ for Pb, 5s^2^5p^5^ for I, 2s^2^2d^2^ for C, 2s^2^2d^3^ for N, and 1s^1^ for H.

For the ground-state calculations of (PEA)_2_PbI_4_ (Pyr-C_2_)_2_PbI_4_ and (Pyr-C_4_)_2_PbI_4_, the Kohn–Sham orbitals are expanded in a basis of plane waves up to a cutoff of 90 Ry. The Brillouin zone is sampled using a uniform, unshifted mesh of 2 × 3 × 3 *k*-points comprising 8 irreducible points. For (PEA)_2_PbI_4_, an unshifted mesh of 1 × 3 × 3 *k*-points comprising 4 irreducible points is used instead, since the unit cell of this system contains two perovskite layers instead of one.

We obtain quasiparticle (QP) energies by using Green’s function-based many-body perturbation theory in the *GW* approximation^113,114^ as implemented in the BerkeleyGW software package.^115^ For this, the DFT-PBE orbitals and eigenvalues are used to construct the zeroth-order, one-particle Green’s function *G*_*0*_ and the screened Coulomb interaction *W*_*0*_. Spin–orbit coupling (SOC) is included self-consistently in all calculations unless otherwise noted.^115^

The frequency dependence of the screened Coulomb interaction is modeled using the generalized plasmon-pole model of Godby and Needs.^116^ We use plane-wave cutoffs of 8 Ry for constructing the polarizability and the screened Coulomb interaction, including 1950, 2100, and 2000 empty bands for (PEA)_2_PbI_4_, (Pyr-C_2_)_2_PbI_4_, and (Pyr-C_4_)_2_PbI_4_, respectively. With these settings, the band gaps are converged to within 0.1 eV (see Figure S6).

For these calculations, we use the (PEA)_2_PbI_4_ relaxed crystal structures obtained from ref 111.^117^ and (Pyr-C_2_)_2_PbI_4_ and (Pyr-C_4_)_2_PbI_4_ experimental structures obtained from refs 7 and 44, respectively. Additionally, we have relaxed the experimental (Pyr-C_4_)_2_PbI_4_ structure using DFT-PBE with the Tkatchenko-Sheffler method for dispersion corrections. A comparison of DFT-PBE band structures using the relaxed and experimental crystal structures is shown in Figure S8 and demonstrates that geometry optimization does not change the band ordering of (Pyr-C_4_)_2_PbI_4_ qualitatively and leads to a decrease of the DFT-PBE band gap by only 125 meV. It is of note that the experimental crystal structure for (Pyr-C_2_)_2_PbI_4_ ^8,118^ used for our calculations is marginally different than the one measured in this work. To confirm that no qualitative changes regarding band alignment or hybridization are present between these two structures, we compared the two band structures but observed no qualitative differences (Figure S10).

For determining the effective mass tensor, we calculate the second derivatives of the valence and conduction band edges with respect to the wave vector **k** along the three crystallographic directions.

$$\frac{1}{{m^{*}}_{\alpha \beta }}=\frac{1}{\hbar^{2}}\frac{\partial^{2}\varepsilon }{\partial k_{\alpha }\partial k_{\beta }}$$

To obtain these band edge curvatures, an interpolated *G*_0_ *W*_0_@PBE+SOC energy correction is obtained by using the inteqp function included in the BerkeleyGW package. For this, the electron self-energy correction is expanded in a set of 100 valence and 100 conduction bands on a 2 × 3 × 3 grid and interpolated to a regular grid of points around the γ point.

These second-order partial derivatives are central finite differences in the first order. The effective mass tensor is then diagonalized for the conduction and valence bands to obtain the electron and hole effective masses, respectively. Then, we calculate the isotropic hole and electron effective masses as the harmonic mean m*, of the x and y masses of the valence and conduction band effective masses, respectively.

$$m^{*}=\frac{2m_{x}m_{y}}{m_{x}+m_{y}}$$

For calculating linear optical absorption spectra, exciton binding energies, and exciton wave functions, we solve the Bethe-Salpeter Equation (BSE),^113,119^ within the Tamm-Dancoff approximation. For (Pyr-C_2_)_2_PbI_4_ and (Pyr-C_4_)_2_PbI_4_, we expand the electron–hole interaction kernel in a set of 32 valence and 32 conduction bands on the 2 × 3 × 3 grid and interpolate it to a 4 × 12 × 12 grid using 16 valence and 16 conduction bands. For (PEA)_2_PbI_4_, the same is done on the 1 × 3 × 3 grid. With these settings, the energy of the first excited state of both systems is converged to within 50 meV (see Figure S22).

To visualize the excitonic wave function of excitation *s*, we consider its expansion in terms of vertical electron–hole transitions

$$\Psi^{s}\left(r_{e},r_{h}\right)=\sum_{\mathrm{cvk}}A_{\mathrm{vck}}^{s}\psi_{\mathrm{ck}}\left(r_{e}\right)\psi_{\mathrm{vk}}^{*}\left(r_{h}\right)$$

This correlated six-dimensional exciton wave function describes the correlated spatial distribution of the electron and hole. For visualization, we fix the position of the hole at *r*_*h*_ and examine the resulting distribution function

$$\Psi^{s}\left(r;r_{h}\right)=\sum_{\mathrm{cvk}}A_{\mathrm{vck}}^{s}\psi_{\mathrm{ck}}\left(r_{e}=r_{h}+r\right)\psi_{\mathrm{vk}}^{*}\left(r_{h}\right)$$

where *r* denotes the relative electron–hole distance at fixed *r*_*h*_. A sufficiently large supercell is required to capture the spatial extend of the excitonic wave function. Additionally, sampling of different hole positions may be needed to fully characterize the spatial distribution of the exciton.

### X-ray Diffraction

Powder X-ray diffraction (XRD) measurements were carried out at ambient temperature on a Bruker D8 goniometer equipped with a Göbel mirror and a 1D lynxeye detector using Cu Kα radiation.

### Single-Crystal X-ray Structure Determination

For the structure of (Pyr-C_2_)_2_PbI_4_ (code **EGSC004)**, X-ray intensity data were collected at 100 K, on a Rigaku Oxford Diffraction Supernova Dual Source (Cu at zero) diffractometer equipped with an Atlas CCD detector using ω scans and Mo Kα (λ = 0.71073 Å) radiation. The images were interpreted and integrated with the program CrysAlisPro.^120^ Using Olex2,^121^ the structure was solved with the ShelXT^122^ structure solution program using Intrinsic Phasing and refined by full-matrix least-squares on F^2^ using the ShelXL program package.^123^ Non-hydrogen atoms were anisotropically refined and the hydrogen atoms in the riding mode with isotropic temperature factors fixed at 1.2 times U­(eq) of the parent atoms. The data showed nonmerohedral twinning and the structure was refined with a twin fraction of 0.29504.

#### Crystal Data for Compound (Pyr-C_2_)_2_PbI_4_

C_36_H_32_I_4_N_2_Pb, *M* = 1207.42, monoclinic, space group *P*2_1_/c (No. 14), *a* = 25.1469(8) Å, *b* = 7.9262(3) Å, *c* = 8.7499(3) Å, β = 92.216(3)°, *V* = 1742.70(11) Å^3^, *Z* = 2, *T* = 100 K, ρ_calc_ = 2.301 g cm^–3^, μ­(Mo–Kα) = 8.411 mm^–1^, *F*(000) = 1112, 18035 reflections measured, reflections were not merged because of nonmerohedral twinning, and were used in all calculations. The final *R*1 was 0.0445 (*I* > 2σ (*I*)) and *w*R** 2 was 0.1029 (all data).

### Steady-State Optical Hyperspectral Microscopy

Wide-field, hyperspectral photoluminescence (PL), transmittance (*T*) and reflectance (*R*) microscopy measurements were conducted using a Photon etc. IMA system. Olympus NA = 0.9 objective lens, with 100× magnification was used for all measurements. To correct for aberrations stemming from the optical elements in the system, the apparatus was calibrated using a reference sample to determine the postprocessing parameters that will be used to correct the image distortion. Chromatic aberrations were mitigated by automatically changing the *z* position (focus) of the sample for every collected central wavelength based on a previously performed calibration measurement. To minimize oxygen and humidity-related degradation behavior, samples were stored in a nitrogen-filled glovebox until immediately prior to measurement. A 405 nm continuous wave laser served as the excitation source for the PL measurements and a broadband halogen lamp was used for *R* and *T* microscopy measurements. The excitation laser was filtered by a high quality 405 nm Semrock dichroic mirror to separate the photoluminescence and excitation signals. The emission from the sample was incident on a volume Bragg grating, which spectrally split the light onto a silicon Hamamatsu CMOS camera with 2048 × 2048 pixel array.

A calibrated white light lamp was coupled to an integrating sphere, through the objective lens, to establish the system’s relative sensitivity in the PL detection optical path both spectrally and spatially. The measured lamp spectrum at each point was compared to the lamp’s known spectrum. This enabled spectral calibration of the detected photoluminescence. A Jacobian conversion is applied to convert between the recorded wavelength domain and energy domain.

To determine local reflectance, the macroscopic reflectance spectrum of a calibration mirror was measured. Before each hyperspectral measurement of a given perovskite sample, a hyperspectral measurement of the calibration mirror was also taken. A spatial median filter was applied to these data to reduce the influence of local imperfections in the mirror. The reflected spectrum from the mirror at each point was divided by the macroscopic reflectance spectrum to obtain the calculated incident lamp spectrum. Once a sample had been measured in reflection, the data were divided by this lamp spectrum to obtain the reflectance of the sample.

For polarization resolved hyperspectral *R* measurements, a linear broadband polarizer was inserted in the lamp excitation path. The orientation of the polarizer was changed to study the effects of the polarization anisotropy. To probe OOP optical transitions, the polarization axis was colinear with the *z* axis ([001] axis) of the single crystal. To probe the IP optical transitions, the polarization axis was colinear with [100] (*x*) or [010] (*y*) axes. This was achieved by physically rotating the crystal in all three orthogonal directions while controlling the orientation of the linear polarization axis in *x*,*y* plane.

### Ultraviolet–Visible Absorption Spectroscopy

A Shimadzu UV–vis-NIR spectrophotometer UV-3600Plus was used to record UV–vis absorption spectra of the spin-coated polycrystalline films deposited on glass. A glass substrate was used as a blank.

### Photoluminescence Spectroscopy

Photoluminescence (excitation) spectra of polycrystalline thin films on glass substrates were recorded on an FLS1000 with a monochromatic Xe lamp as the excitation source and a photomultiplier tube as a detector.

### Time-Resolved Photoluminescence Spectroscopy

Time-resolved photoluminescence spectra were recorded using an electronically gated iCCD camera (Andor iStar DH740 CCI-010) coupled with a calibrated grating spectrometer (Andor SR303i). The 800 nm output of a Ti:sapphire laser (Spectra Physics Solstice Ace, 7 W, 1 kHz, 100 fs) was used to generate the excitation wavelength of 400 nm through second-harmonic generation (SHG) in a β-barium borate crystal. A 425 nm long-pass filter was inserted before the camera to prevent laser scattering signals in the spectrometer. PL spectra were recorded with gate widths of 5 ns at different iCCD gate delays with respect to the excitation pulse.

### Transient Absorption Spectroscopy

Picosecond transient absorption (TA) spectroscopy measurements for (Pyr-C_4_)_2_PbI_4_ and (PEA)_2_PbI_4_ polycrystalline thin films were performed on a home-built setup. A broad ultraviolet–visible spectrum spanning 350 to 700 nm was produced by pumping a CaF_2_ crystal with a focused beam output from an 800 nm Ti:sapphire laser (Spectra Physics Solstice Ace, 7 W, 1 kHz, 100 fs) followed by collimation. This same output beam was used to generate a 400 nm pump wavelength through second-harmonic generation (SHG) in a β-barium borate crystal. The pump beam is focused weakly onto the sample to generate a near-Gaussian (FWHM ∼500 μm) exciton carrier distribution. A mechanically delayed probe beam is focused to ∼100 μm in a noncollinear fashion and is transmitted through the sample at various pump–probe temporal delays, after which the pulse is dispersed via a grating in a spectrometer (Andor, SR1-ASM-0020) and recorded with a monochrome line scan camera (JAI, SW-4000M-PMCL), which acquires at 1 kHz. The Δ*T*/*T*(λ,*t*) spectra are calculated by subsequently recording *T*_pump,on_(λ,*t*) and *T*_pump,off_(λ,*t*) spectra through pump modulation at 500 Hz using a mechanical chopper.

The TA spectra for (Pyr-C_2_)_2_PbI_4_ were recorded on a commercial TA system (Light Conversion, HARPIA). Here, the probe spectrum is generated by focusing the SH (515 nm) of the 1030 nm Yb:KGW amplifier laser output (Light Conversion Pharos, 5 W, 10 kHz, 200 fs) onto a sapphire crystal. The pump wavelength of 400 nm is generated by a commercial next-generation optical parametric amplifier (Light Conversion, ORPHEUS-Neo).

### Transient Absorption Microscopy

The design of the home-built TAM setup is described elsewhere.^82^ The pump and probe pulses are generated via SHG in two separate β-barium borate (BBO) crystals by propagating a broadband pulse (750–1030 nm) through a 50–50 beam splitter. The broadband pulse is generated via noncollinear optical parametric amplification (NOPA) of a 1030 nm-seeded YAG-based white light continuum (WLC) by the SH (515 nm) of the 1030 nm Yb:KGW amplifier laser output (Light Conversion Pharos, 5 W, 200 kHz, 200 fs), followed by compression using a combination of chirped mirrors and wedge prisms (Layertec). Spectral tuning of the probe pulse was achieved through rotation of the BBO crystal axis and incident pump angle, yielding a 480 nm centered spectrum for (Pyr-C_2_)_2_PbI_4_ and 500 nm for (Pyr-C_4_)_2_PbI_4_. To probe at longer wavelengths required for (PEA)_2_PbI_4_ the 1030 nm laser output is focused on a sapphire crystal to generate a visible broadband (500–650 nm) spectrum, followed by collimation and compression. Pump pulses of 450 nm were used to selectively excite the lowest energy exciton.

The pump pulse is focused through an oil-immersed objective to produce a near-diffraction limited (FWHM ∼270 nm, σ ∼ 110 nm for λ = 450 nm) local exciton carrier density onto the crystalline flakes. A mechanically delayed and counter-propagating collinear wide-field probe (FWHM ∼50 μm) is then transmitted through the sample at various pump–probe temporal delays and imaged onto an emCCD camera (Rolera Thunder, QImaging) via a 250× magnifying objective-imaging lens system. The Δ*T*/*T*(*x*,*y*,*t*) images are calculated by subsequently recording *T*_pump,on_(*x*,*y*,*t*) and *T*_pump,off_(*x*,*y*,*t*) images through pump modulation at 40 Hz using a chopper.

## Supplementary Material



## Data Availability

The data that support the findings of this study is available to download at the University of Cambridge’s Apollo Repository [DOI: 10.17863/CAM.120583].

## Notes

Additional experimental details, synthetic procedures, ^1^H NMR and ^13^C NMR spectra, DFT calculations, XRD patterns, UV–vis spectra, PL spectra, TAM images and kinetics (PDF)
