# Supplementary material for: Molecular Engineering of Interlayer Exciton Delocalization in 2D Perovskites
Source: J Am Chem Soc. 2025 Aug 21;147(35):31541–57. doi: 10.1021/jacs.5c05621 (PMC12412180; doi:10.1021/jacs.5c05621)
Supplement: Supplementary file 1 [file ja5c05621_si_001.pdf]

Supporting information: Molecular engineering of interlayer exciton delocalization in 2D perovskites

Authors: Yorrick Boeije[1,2], Fabian Lie[3], Miloš Dubajić[1], Ediz Garip[4], Arthur Maufort[4], Raisa-Iona Biega[3], Stijn Lenaers[4], Mylène Sauty[2], Pratyush Ghosh[2], Aleksandar Radić[2], Amélie Loher[5], Paola La Magna[7], Hayden Salway[1], Arjun Ashoka[2], Xian Wei Chua[1,2], Qichun Gu[1], Kristof Van Hecke[7], Laurence Lutsen[4,6], Dirk Vanderzande[4,6], Akshay Rao[2], Wouter T. M. Van Gompel[4,6]*, Linn Leppert[3]*, Samuel D. Stranks[1,2]*

Affiliations:

[1]Department of Chemical Engineering and Biotechnology, University of Cambridge; Cambridge, CB3 0AS, UK.

[2]Department of Physics, Cavendish Laboratory, University of Cambridge; Cambridge, CB3 0HE, UK.

[3]MESA+ Institute for Nanotechnology, University of Twente; Enschede, 7522 NH, The Netherlands.

[4]Hasselt University, Institute for Materials Research (imo-imomec), Hybrid Materials Design (HyMaD), Martelarenlaan 42, B-3500 Hasselt, Belgium.

[5]Department of Pure Mathematics and Mathematical Statistics, University of Cambridge; Cambridge, CB3 0WB, UK.

[6]Energyville, imo-imomec, Thor Park 8320, B-3600 Genk, Belgium.

[7]XStruct, Department of Chemistry, Ghent University, Krijgslaan 281-S3, B-9000 Ghent, Belgium.

*Corresponding authors. Email: sds65@cam.ac.uk, l.leppert@utwente.nl, wouter.vangompel@uhasselt.be

Synthesis of ammonium salts

Synthesis of 2-(pyren-1-yl)ethylammonium iodide ($PyrC_2NH_3I$)

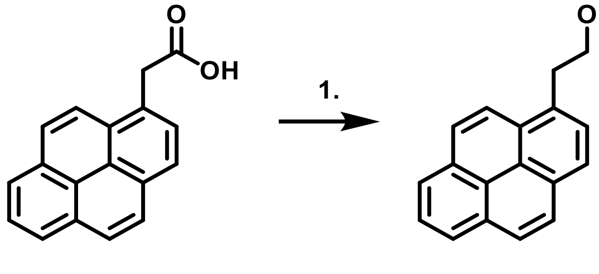

I II

2-(pyren-1-yl)ethan-1-ol (II): $LiAlH_4$ (1.09 g, 28.8 mmol, 1.5 eq) was dispersed in dry THF (70 mL) under N_2 atmosphere. After heating the dispersion to 85 °C a solution of 1-pyreneacetic acid (**I**, 5 g, 19.2 mmol) in THF (30 mL) was added dropwise to the refluxing mixture. The reaction was continued for 2 h, after which a saturated K_2CO_3 solution was added slowly to the reaction mixture. Next, the mixture was extracted with 1M HCl (1 x 100 mL) and EtOAc (3 × 150 mL). Then, the organic layer was dried with magnesium sulfate, filtered and the solvent was removed by vacuum distillation. The residue was purified by flash chromatography (DCM), to obtain **II** as a light yellow solid (3.99 g, 84%).

^{1}H NMR (400 MHz, Chloroform-*d*) δ 8.30 (d, *J* = 9.3 Hz, 1H), 8.17 (dq, *J* = 7.9, 1.2 Hz, 2H), 8.15 – 8.09 (m, 2H), 8.03 (s, 2H), 8.02 – 7.97 (m, 1H), 7.90 (d, *J* = 7.8 Hz, 1H), 4.09 (t, *J* = 6.7 Hz, 2H), 3.62 (t, *J* = 6.7 Hz, 2H).

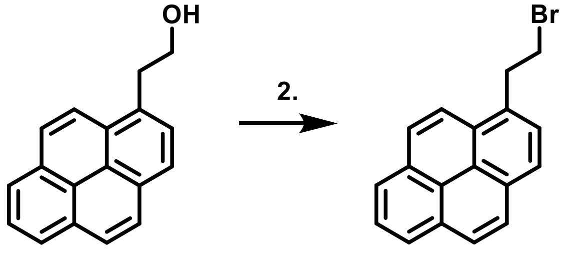

II III

1-(2-bromoethyl)pyrene (III): CBr_4 (6.71 g, 20.24 mmol, 1.25 eq), K_2CO_3 (3.36 g, 24.29 mmol, 1.5 eq) and **II** (3.99 g, 16.19 mmol) were dissolved in DCM (160 mL) under a N_2 atmosphere and cooled to 0 °C. A solution of Ph_3P (5.31 g, 20.24 mmol, 1.25 eq) in DCM (50 mL) was added dropwise to the stirring mixture which was subsequently allowed to slowly warm up to room temperature. Stirring continued for an additional 10 h, after which the reaction mixture was filtered and the filtrate was concentrated. The crude product was further purified by flash chromatography (2:8 DCM:*n*-hexane) and recrystallization from DCM/MeOH, to obtain **III** as light yellow needles. (4.11 g, 82%).

^{1}H NMR (400 MHz, Chloroform-*d*) δ 8.23 (d, *J* = 9.2 Hz, 1H), 8.21 – 8.17 (m, 2H), 8.17 – 8.11 (m, 2H), 8.05 (d, *J* = 1.6 Hz, 2H), 8.03 – 7.98 (m, 1H), 7.89 (d, *J* = 7.8 Hz, 1H), 3.90 (ddd, *J* = 8.7, 7.2, 1.2 Hz, 2H), 3.77 (ddd, *J* = 8.3, 7.2, 1.1 Hz, 2H).

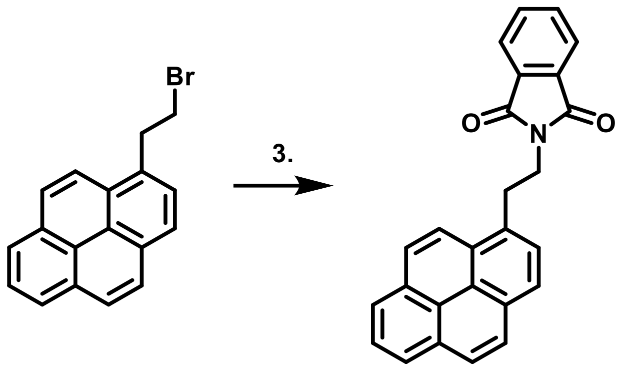

III IV

2-(2-(pyren-1-yl)ethyl)isoindoline-1,3-dione (IV): potassium 1,3-dioxoisoindolin-2-ide (1.85 g, 9.96 mmol, 1.5 eq) and **III** (2.05 g, 6.64 mmol) were dissolved in DMF (50 mL), heated to 80 °C and stirred for 6 h. After the reaction was completed, 100 ml $CHCl_3$ was added and the reaction mixture was extracted with a saturated solution of NH_4Cl (5x 150 mL). Then, the organic layer was dried with magnesium sulfate, filtered and the solvent was removed by vacuum distillation. The crude was further purified by column chromatography (6:4 DCM:*n*-hexane). Product **IV** was obtained as a bright yellow solid (1.97 g, 79%).

^{1}H NMR (400 MHz, DMSO-d_6) δ 8.43 (d, *J* = 9.3 Hz, 1H), 8.28 – 8.23 (m, 2H), 8.21 (d, *J* = 9.2 Hz, 1H), 8.15 (d, *J* = 7.8 Hz, 1H), 8.10 (d, *J* = 2.0 Hz, 2H), 8.04 (t, *J* = 7.6 Hz, 1H), 7.87 (d, *J* = 7.8 Hz, 1H), 7.82 – 7.75 (m, 4H), 3.99 (dd, *J* = 8.2, 6.6 Hz, 2H), 3.64 (dd, *J* = 8.3, 6.5 Hz, 2H).

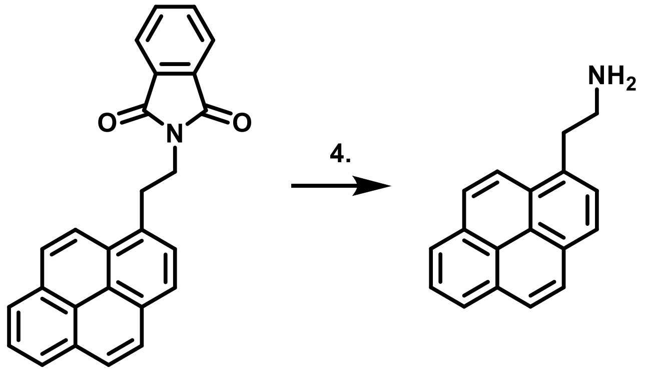

IV V

2-(pyren-1-yl)ethan-1-amine (V): Hydrazine monohydrate (65 wt. % in H_2O, 5.46 mL, 12.25 mmol, 30 eq) was added to a refluxing solution of **IV** (1.41 g, 3.75 mmol) in ethanol (40 mL). Reaction continued for 24 h during which large white crystals precipitated. After the reaction was completed, the reaction mixture was allowed to cool down to room temperature, the precipitate was filtered off, and the solvent was removed by vacuum elimination. Next, the residue was dissolved in chloroform and the filtration process was repeated. The filtrate was concentrated by rotary evaporation to obtain **V** as a dark yellow oil (0.86 g, 93%).

^{1}H NMR (400 MHz, DMSO-d_6) δ 8.37 (d, *J* = 9.2 Hz, 1H), 8.22 (ddd, *J* = 7.8, 5.3, 1.2 Hz, 2H), 8.20 – 8.14 (m, 2H), 8.08 (d, *J* = 2.1 Hz, 2H), 8.01 (t, *J* = 7.6 Hz, 1H), 7.91 (d, *J* = 7.8 Hz, 1H), 3.40 – 3.33 (m, 2H), 2.96 – 2.89 (m, 2H).

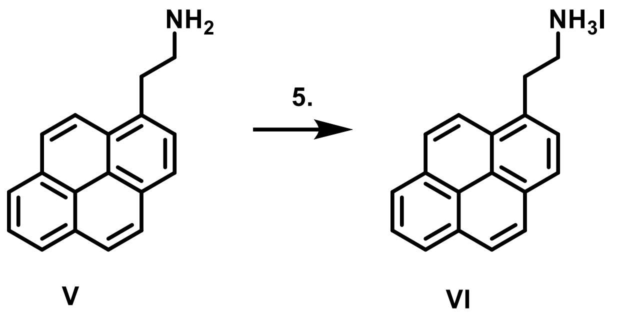

2-(pyren-1-yl)ethan-1-ammonium iodide (VI): HI (57%, unstabilized) was extracted (3x 9:1 $CHCl_3$:tributyl phosphate) to remove impurities. Immediately after extraction 0.48ml (3.67 mmol, 1.05 eq) of this HI was added to a solution of **V** (0.86 g, 3.49 mmol) in EtOH (30 ml). The reaction mixture was stirred at room temperature for 30 minutes while protected from the light. Afterwards, the crude product was purified by dropwise addition of the reaction mixture to 700 mL of ice-cooled diethylether. The resulting precipitate was filtered and washed with additional diethyl ether to yield the ammonium salt **VI** as a pure white solid (1.12g, 86%).

^{1}H NMR (400 MHz, DMSO-d_6) δ 8.37 (d, J = 9.2 Hz, 1H), 8.33 – 8.20 (m, 4H), 8.13 (s, 2H), 8.05 (t, J = 7.6 Hz, 1H), 7.96 (d, J = 7.8 Hz, 1H), 3.65 – 3.53 (m, 2H), 3.25 – 3.14 (m, 2H).

^{13}C NMR (100 MHz, DMSO-d_6) δ 132.0, 131.4, 130.9, 130.6, 129.0, 128.5, 128.4, 128.0, 127.7, 127.0, 125.9, 125.73, 125.70, 124.8, 124.6, 123.6, 40.9, 31.4.

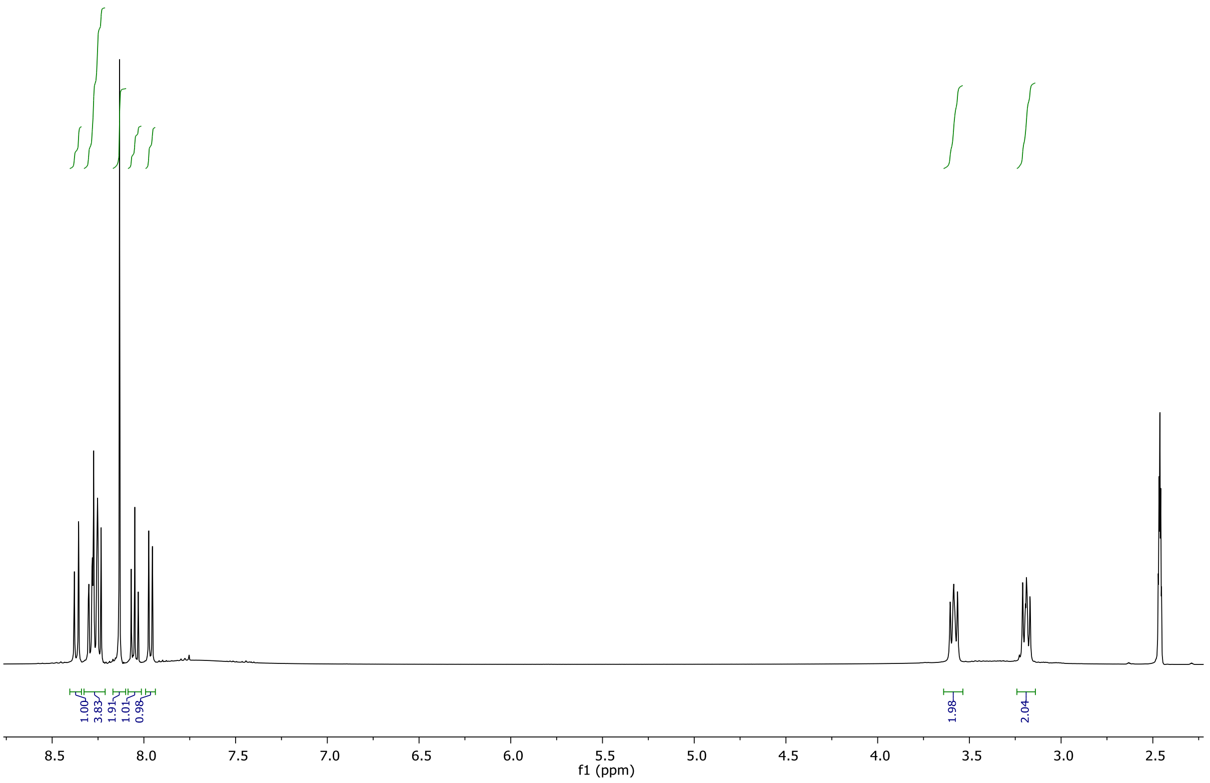

Supplementary Figure S1. ^{1}H NMR spectrum of the $PyrC_2NH_3I$ salt in DMSO-d_6.

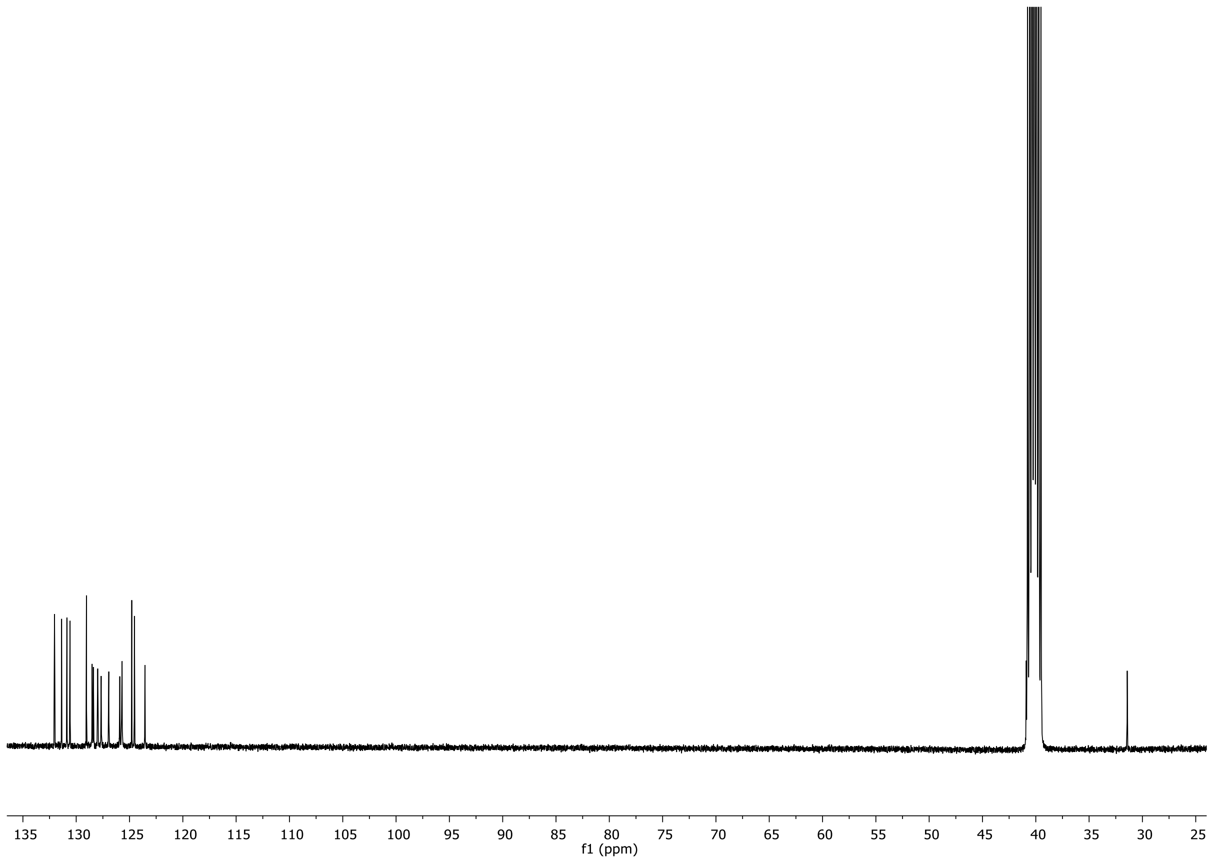

Supplementary Figure S2. ^{13}C NMR spectrum of the $PyrC_2NH_3I$ salt in DMSO-d_6.

Synthesis of 4-(pyren-1-yl)butylammonium iodide ($PyrC_4NH_3I$)

The $PyrC_4NH_3I$ salt was synthesized as described in our earlier work.[1]

Synthesis of phenylethylammonium iodide (PEAI)

2-phenylethylamine (5.000 g, 41.3 mmol) was dissolved in 100 mL chloroform and HI (57 wt%, 5.72 mL, 43.3 mmol) was added. This mixture was stirred in the dark at ambient temperature for 2 h. A thin liquid layer formed on top of the chloroform phase. This layer was carefully isolated and added dropwise to 400 mL diethyl ether, inducing the crystallization of the ammonium salt. Subsequent vacuum filtration and washing with diethyl ether yielded PEAI as colorless crystalline flakes (7.35 g, 72% yield), which were dried under high vacuum and stored in a nitrogen-filled glovebox. ^{1}H NMR (400 MHz, DMSO-d_6) δ 7.76 (s, 3H), 7.37 – 7.30 (m, 2H), 7.28 – 7.21 (m, 3H), 3.10 – 3.01 (m, 2H), 2.86 (dd, J = 9.5, 6.4 Hz, 2H).

Supplementary Table 1. Lattice parameters (a, b and c in Å and α, β, and γ in º), unit cell volume (V in Å^3), average Pb–I bond lengths (in Å) and in-plane tilt angles (δ and ϕ in º) for geometry-optimized $(PEA)_2PbI_4$, $(Pyr\text{-}C_2)_2PbI_4$ and $(Pyr\text{-}C_4)_2PbI_4$.

	$(PEA)_2PbI_4$	$(Pyr\text{-}C_2)_2PbI_4$	$(Pyr\text{-}C_4)_2PbI_4$
**a**	8.669	8.397	8.763
**b**	8.665	7.878	8.704
**c**	32.51	25.27	25.08
**α**	85.73	90.00	90.00
**β**	85.76	94.16	92.65
**γ**	89.22	90.00	90.00
**V**	2428.32	1667.23	1910.67
$d_{Pb\text{–}I,eq}$	3.163	3.015	3.202
$d_{Pb\text{–}I,ax}$	3.253	3.121	3.180
**δ**	13.81	17.14	15.36
**ϕ**	14.90	17.46	15.38

Supplementary Table 2. HOMO and LUMO energies (in eV) of isolated gas-phase molecules calculated with DFT using the B3LYP exchange-correlation functional and using the *GW* approach starting from B3LYP eigenvalues and eigenfunctions and updating the eigenvalues in the construction of *G* and *W* self-consistently. These calculations were carried out using the TURBOMOLE code[2,3] with a def2-TZVPP basis set. Molecular geometries were taken from the experimental crystal structures of $(PEA)_2PbI_4$, $(Pyr\text{-}C_2)_2PbI_4$ and $(Pyr\text{-}C_4)_2PbI_4$, respectively, and geometry-optimized using DFT-B3LYP.

	PEA^+	$Pyr\text{-}C_2^+$	$Pyr\text{-}C_4^+$	PEA	Pyr-C_2	Pyr-C_4
HOMO B3LYP	-9.90	7.61	-7.18	-6.39	-5.45	-5.47
LUMO B3LYP	-5.38	-5.5	-5.52	-0.39	-1.69	-1.72
Gap B3LYP	4.52	2.11	1.65	5.99	3.76	3.75
HOMO GW	-12.72	-9.7	-9.26	-9.1	-7.13	-7.13
LUMO GW	-2.70	-2.86	-2.82	1.57	-0.25	-0.29
Gap GW	10.02	6.83	6.44	10.67	6.88	6.85

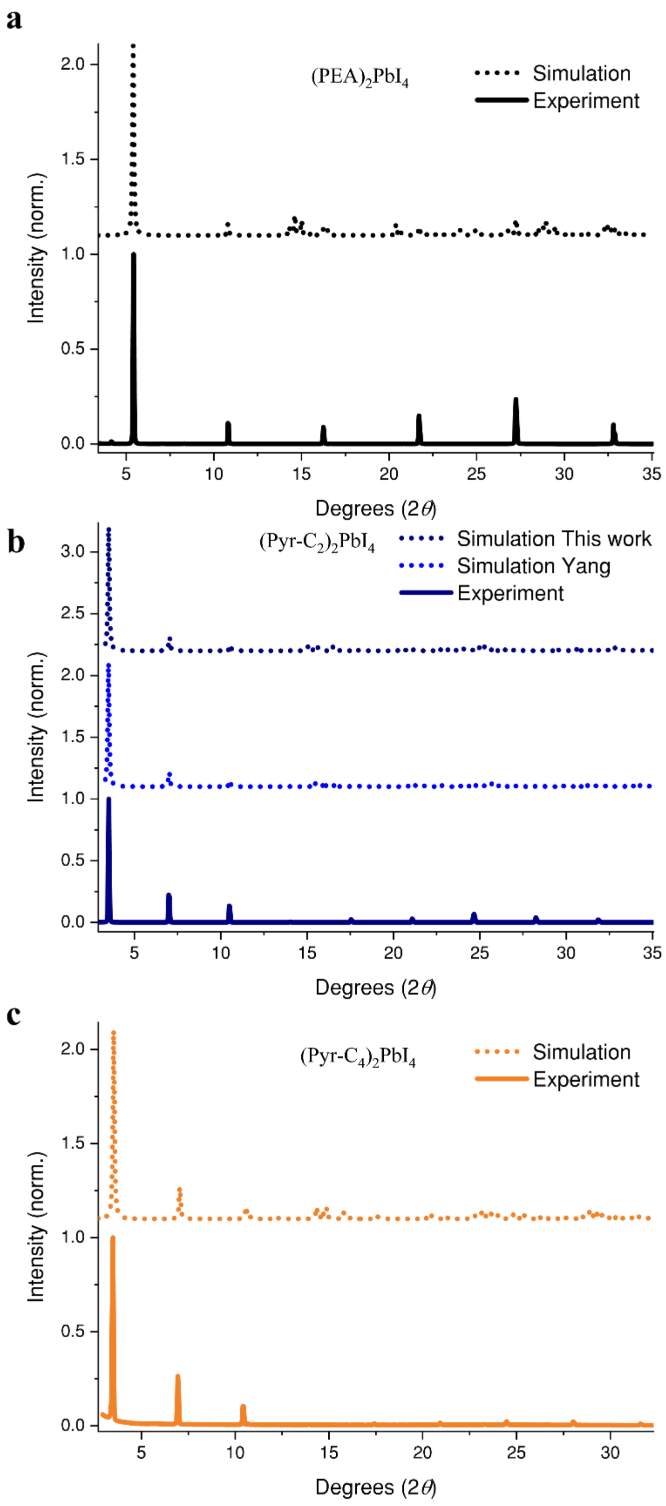

Supplementary Figure S3. X-ray diffraction patterns for single crystals grown on glass using the AVCC method, overlayed with simulated patterns based on the CIF files of experimentally determined crystal structure of $(PEA)_2PbI_4$ (a), $(Pyr\text{-}C_2)_2PbI_4$ (b) and $(Pyr\text{-}C_4)_2PbI_4$ (c), respectively. Note that the crystals grown on glass possess a strong preferential orientation. A simulated pattern from the crystal structure of $(Pyr\text{-}C_2)_2PbI_4$ received from Yang and co-workers is included, showing no discernible differences with our simulated pattern.[4]

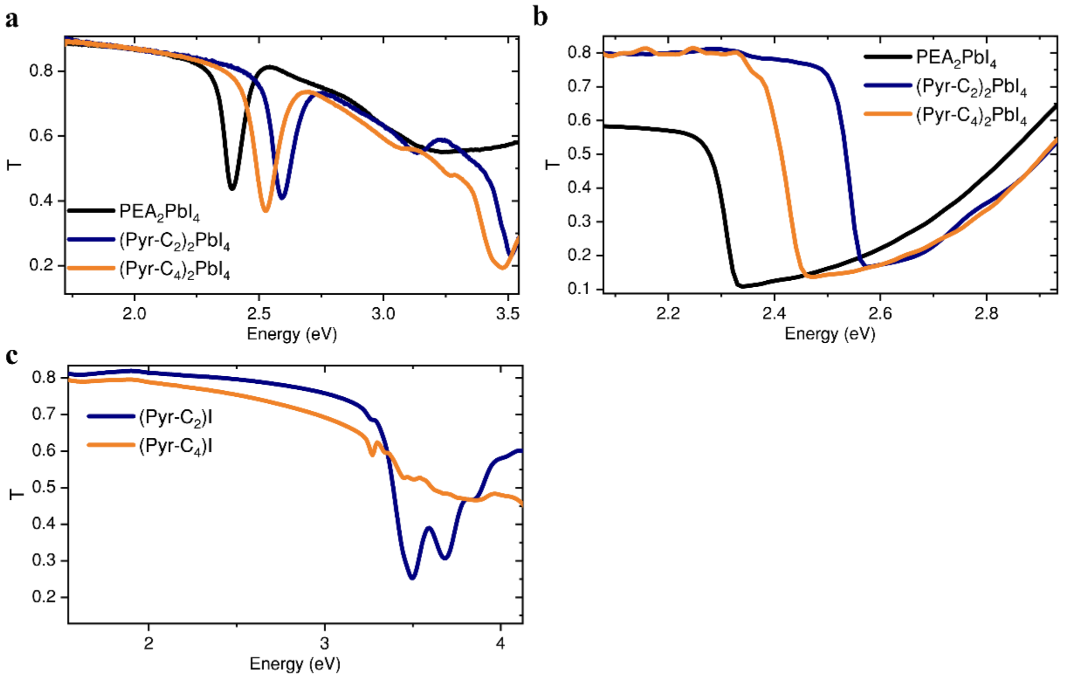

Supplementary Figure S4. Transmission spectra of polycrystalline 2D perovskite thin films (a). Transmission spectra of 2D perovskite single crystals (b). The spectrum becomes inaccurate in the high energy range due to saturation by too strong absorption. Transmission spectra of (Pyr-C_4)I and (Pyr-C_2)I salt thin films (c).

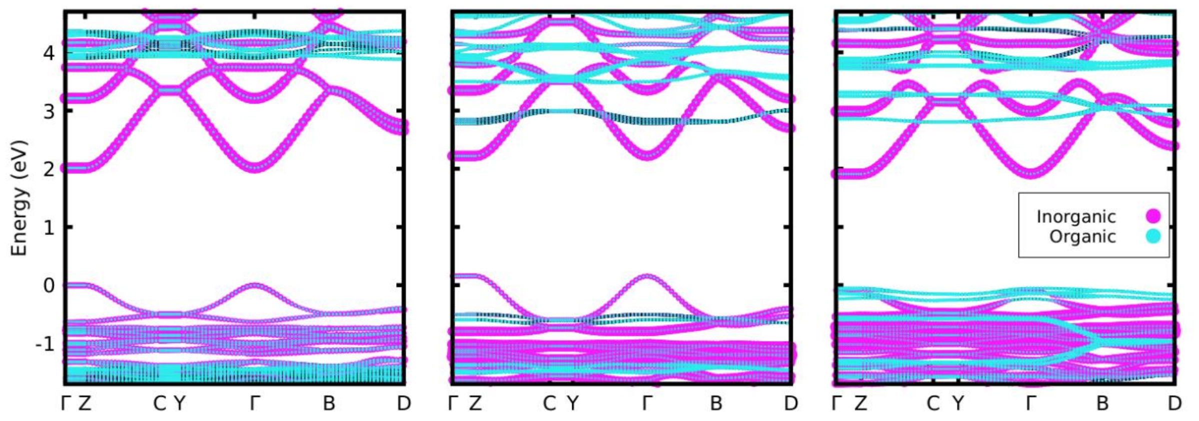

Supplementary Figure S5. Complete DFT-PBE+SOC band structures of $(PEA)_2PbI_4$ (left), $(Pyr\text{-}C_2)_2PbI_4$ (middle) and $(Pyr\text{-}C_4)_2PbI_4$ (right). The bands are shifted and aligned as explained in the caption of Fig. 2 of the main text.

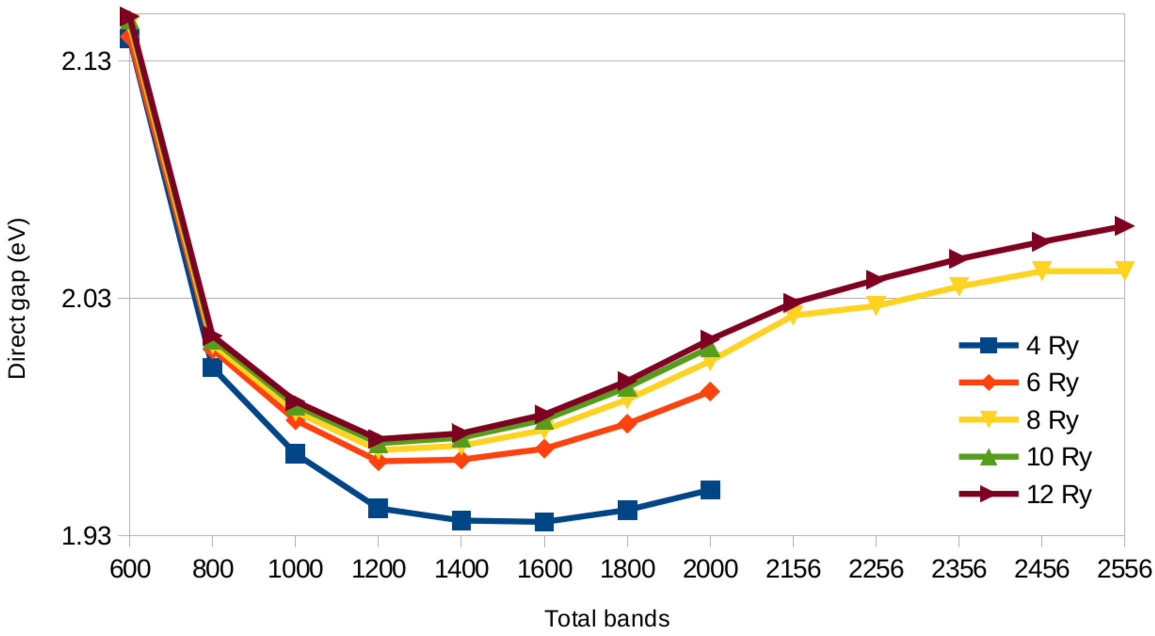

Supplementary Figure S6. Convergence of the direct G_0W_0@PBE+SOC bandgap for $(Pyr\text{-}C_2)_2PbI_4$ with respect to the total amount of states (occupied+empty) included in the calculation of the *GW* self-energy and the energy cutoff for the plane-wave expansion of the screened Coulomb interaction (in Ry).

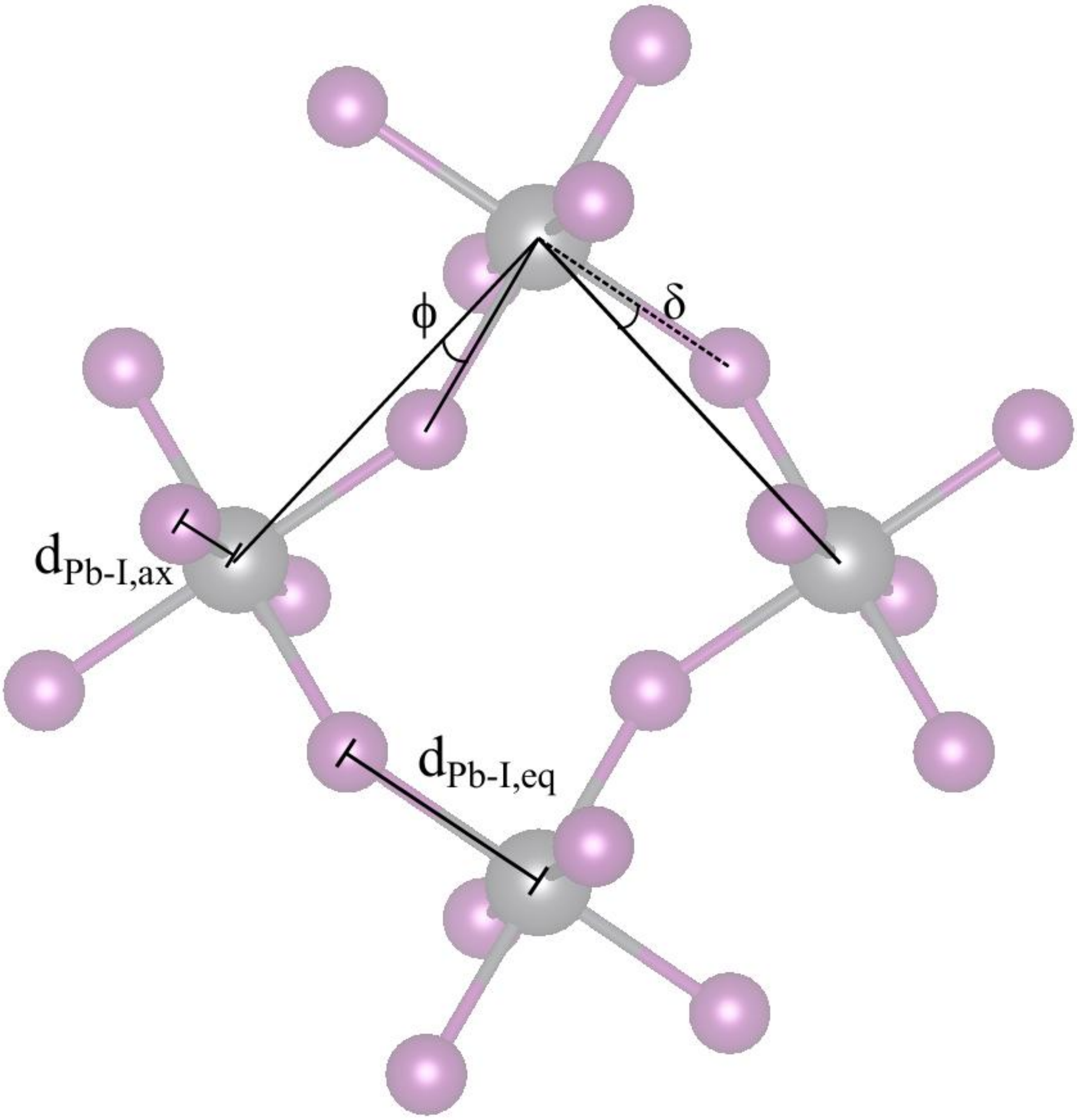

Supplementary Figure S7. Visualization of octahedral distortion parameters (Table S1).

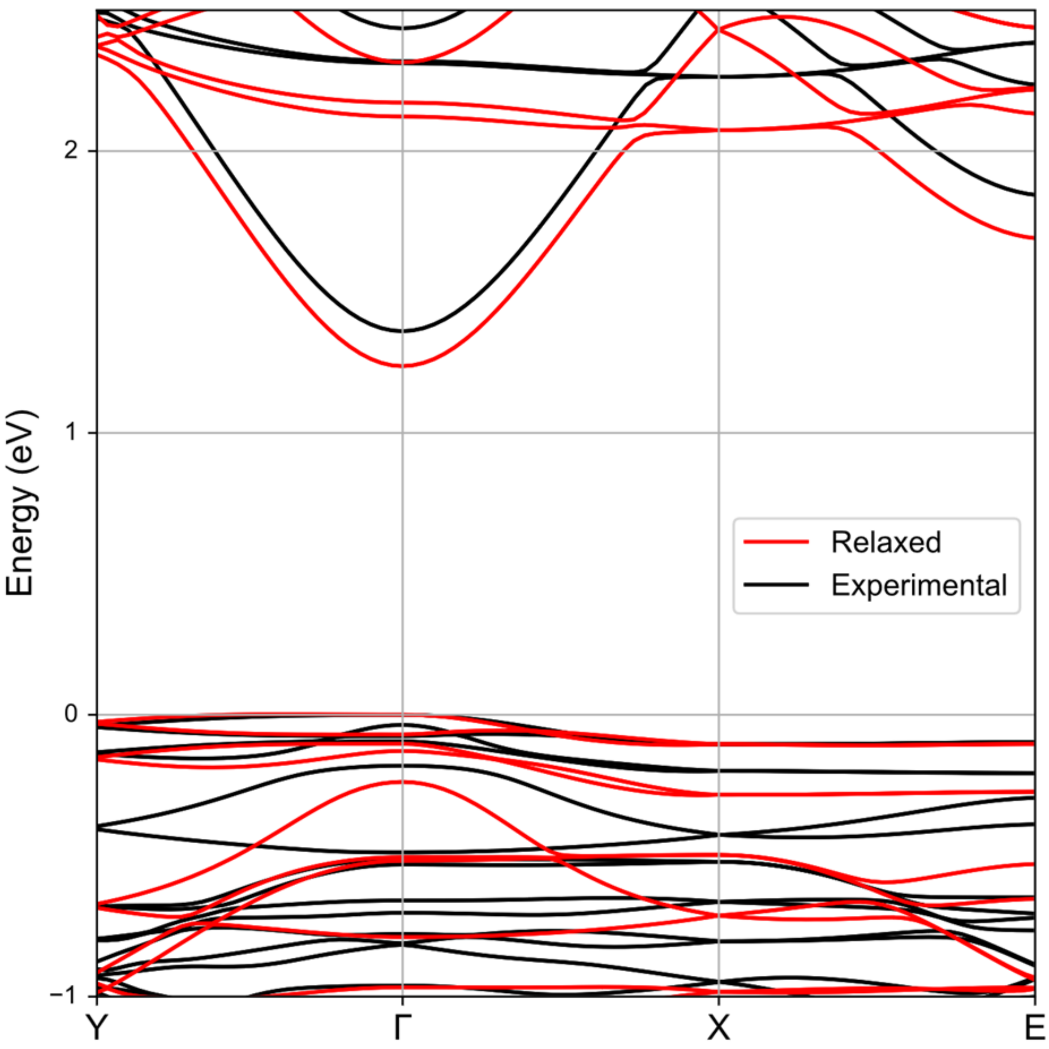

Supplementary Figure S8. Comparison of band structures of relaxed and experimental geometries for $(Pyr\text{-}C_4)_2PbI_4$ calculated using DFT-PBE+SOC.

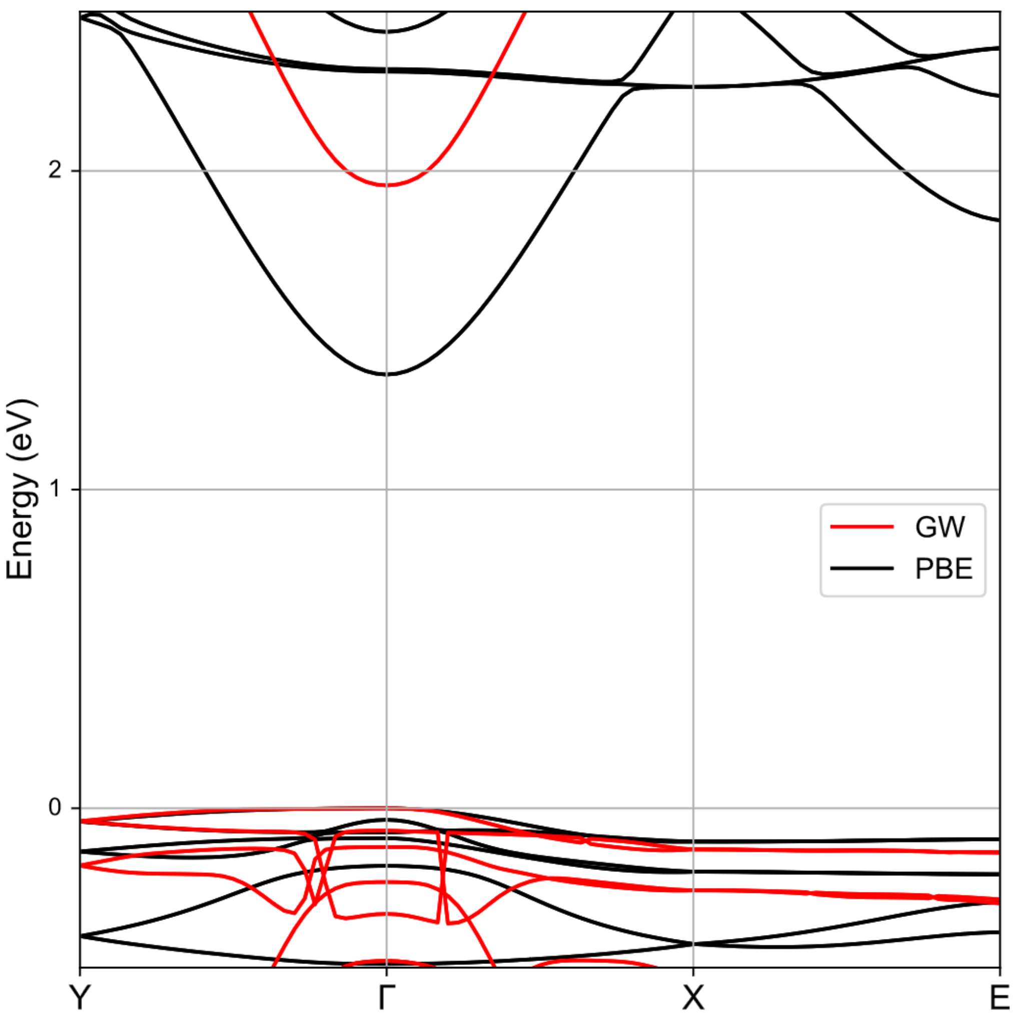

Supplementary Figure S9. Comparison of DFT-PBE+SOC and G_0W_0@PBE+SOC band structures. The *GW* energies are interpolated from a grid of 3x3x1 **k**-points, which causes irregularities in the band structure. However, the states around the lowest direct transition, at the Γ point, are interpolated correctly due to being close to one of the coarse grid points. This illustrates that the band ordering of HOMO and VBM does not change when *GW* quasiparticle corrections are applied.

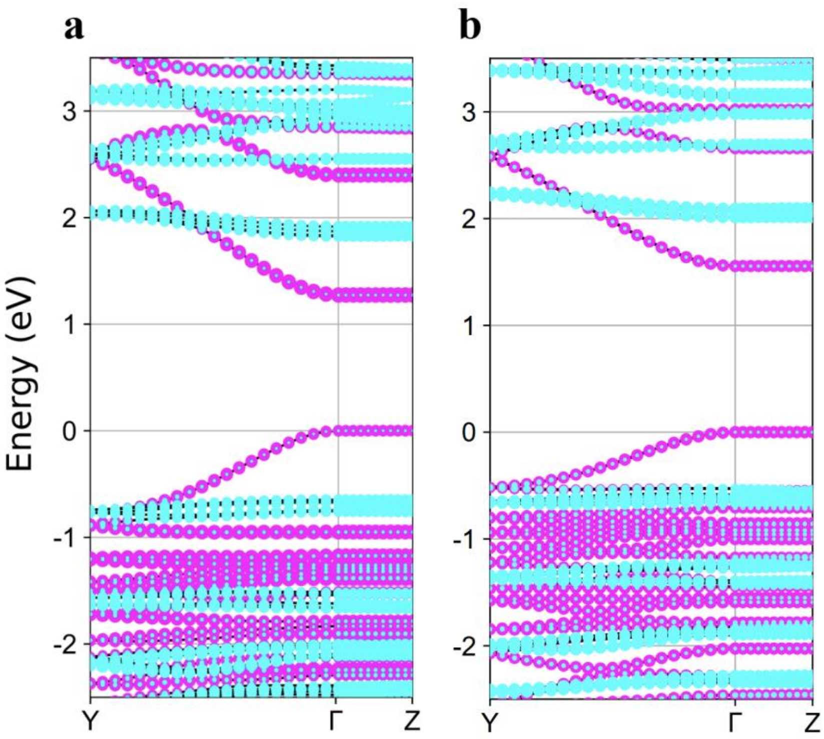

Supplementary Figure S10. DFT-PBE+SOC band-structures for $(Pyr\text{-}C_2)_2PbI_4$ using a structure previously determined by Yang and co-workers (and used for our exciton calculations)[5] (a) and an experimental structure determined in this work (b). The difference in band dispersion in the VBM is due to slight differences in the magnitude of octahedral tilts in these two structures.

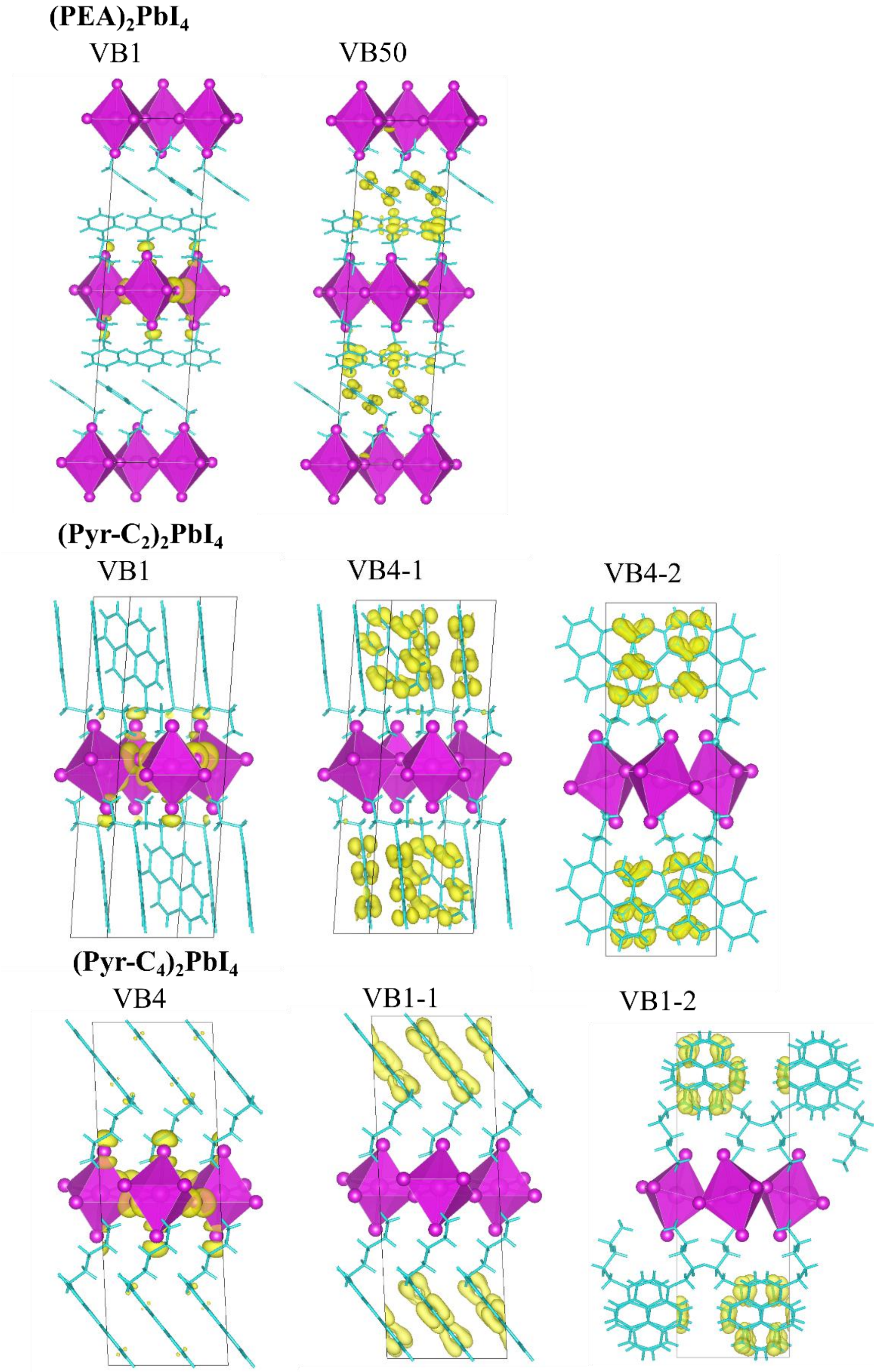

Supplementary Figure S11. Orbital density plots for relevant valence bands (VB). For each material an orbital density localized on the inorganic and the organic sublattice is shown. For (Pyr-C$_2$)$_2$PbI$_4$ and (Pyr-C$_4$)$_2$PbI$_4$ we have included two perspectives for ease of visualization of the orbitals derived from the organic sublattice (VB4 and VB1, respectively). Note the small degree of hybridization of the orbital derived from the inorganic sublattice in (Pyr-C$_4$)$_2$PbI$_4$ (VB4), which is absent in (Pyr-C$_2$)$_2$PbI$_4$ (VB1).

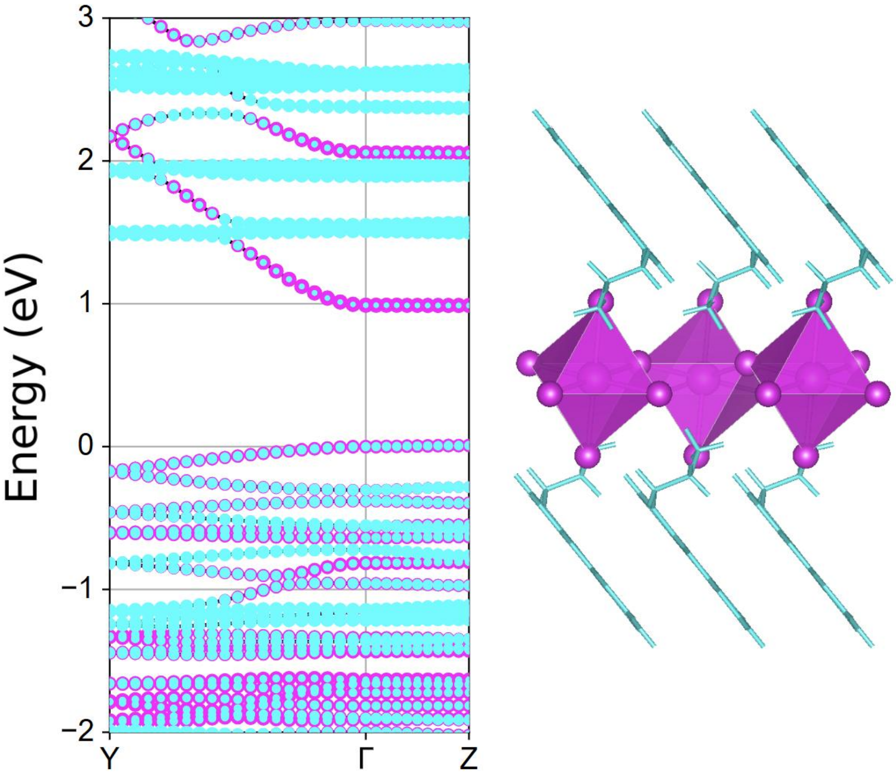

Supplementary Figure S12. DFT-PBE+SOC band-structure of model $(Pyr\text{-}C_2)_2PbI_4$ structure with forced inward tilting of the π-core.

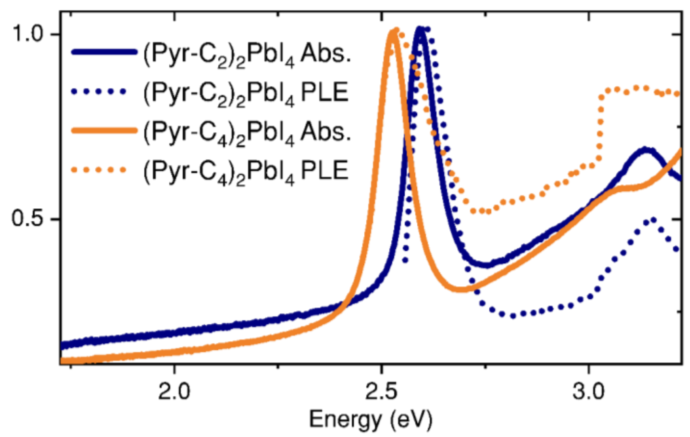

Supplementary Figure S13. Absorption and photoluminescence excitation spectra of polycrystalline thin films. PLE spectra were recorded at 2.5 eV (495 nm) and 2.0 eV (615 nm) for $(Pyr\text{-}C_2)_2PbI_4$ and $(Pyr\text{-}C_4)_2PbI_4$ respectively.

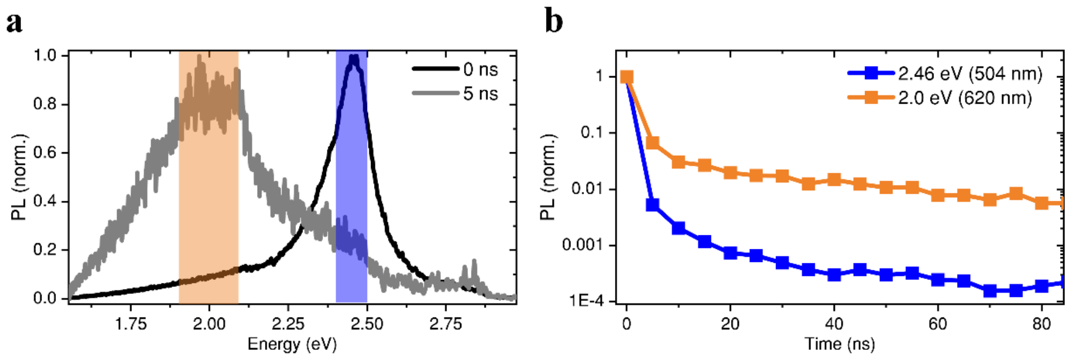

Supplementary Figure S14. Time-resolved photoluminescence spectroscopy on a polycrystalline $(Pyr\text{-}C_4)_2PbI_4$ thin film. λ_{exc} = 400 nm (1 mJ/cm^2). Normalized PL spectra at 0 and 5 ns (a). Normalized PL kinetics at spectral regions indicated in a) (b).

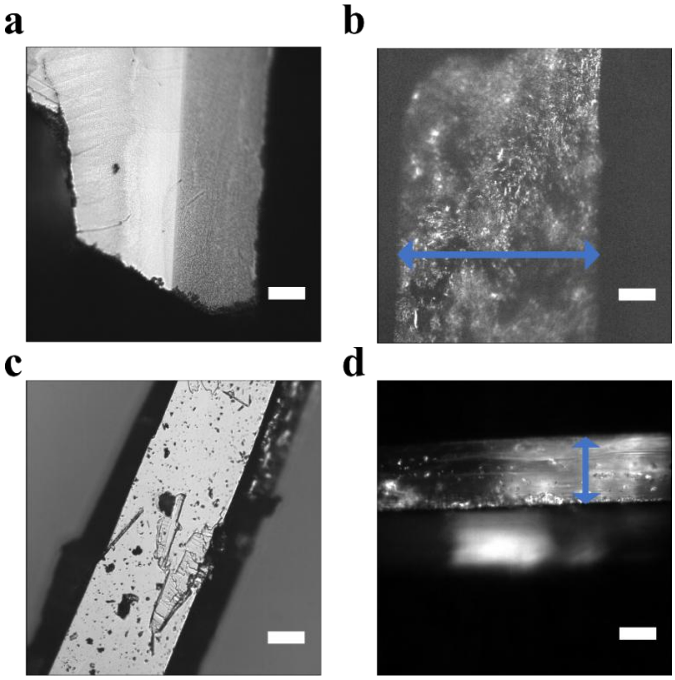

Supplementary Figure S15. Optical images of bulky $(PEA)_2PbI_4$ (a,b) and $(Pyr\text{-}C_4)_2PbI_4$ (c,d) crystals. Panels a and c show plane 1 (lateral view containing both in-plane crystal axes), and panels b and d show plane 2 (cross-sectional view containing the out-of-plane crystal axis). Scale bar indicates 50 μm. Blue arrows indicate crystal thicknesses, 226 μm and 75 μm for $(PEA)_2PbI_4$ and $(Pyr\text{-}C_4)_2PbI_4$, respectively.

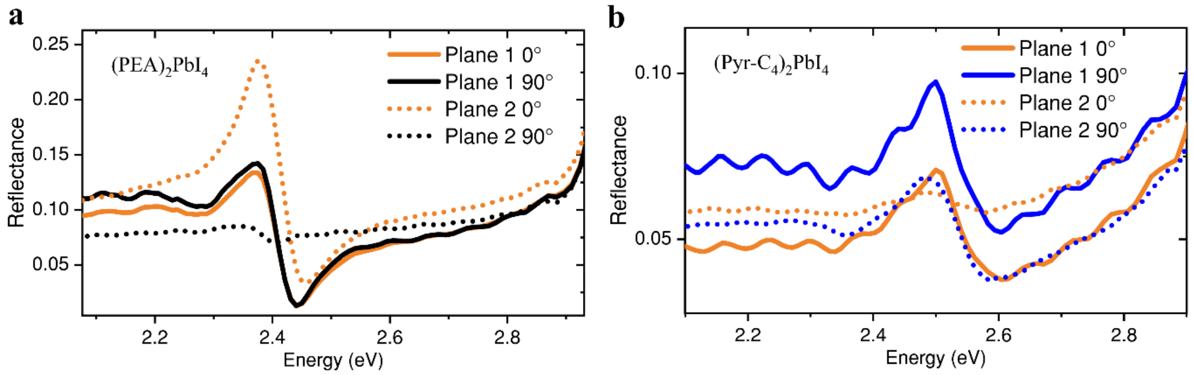

Supplementary Figure S16. 90º and 0º polarized calibrated reflection spectra on both crystal planes 1 and 2 of $(PEA)_2PbI_4$ and $(Pyr\text{-}C_4)_2PbI_4$. Crystal planes are visualized in Fig. 3a of the main text.

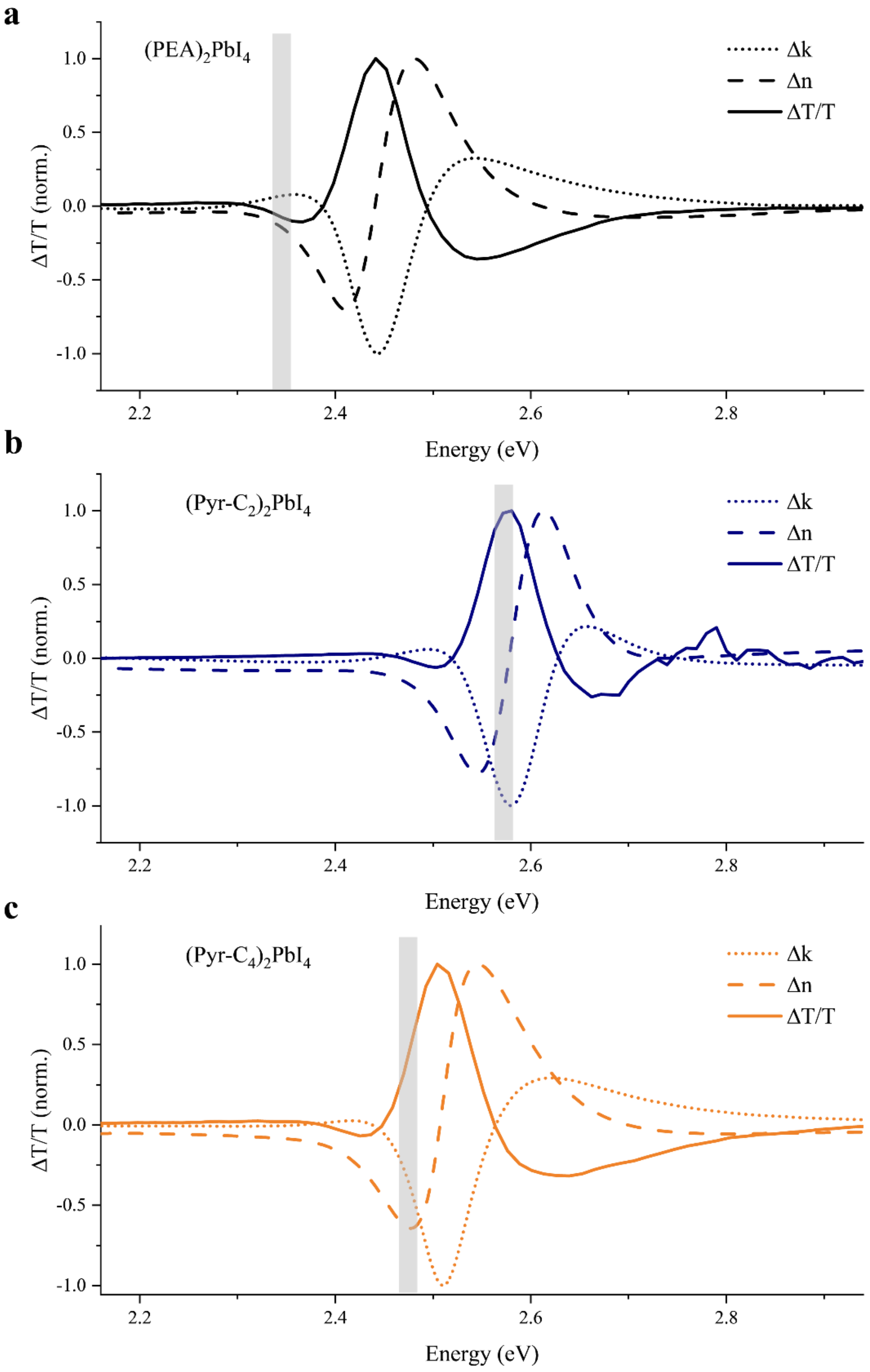

Supplementary Figure S17. KK-transformed transient absorption spectra at 1 ps of polycrystalline films. Grey bars indicate probe energies selected for TAM measurements. Due to the larger thickness of the $(PEA)_2PbI_4$ flakes, the probe was selected further away from the main bleach compared to $(PyrC_2)_2PbI_4$ and $(PyrC_4)_2PbI_4$.

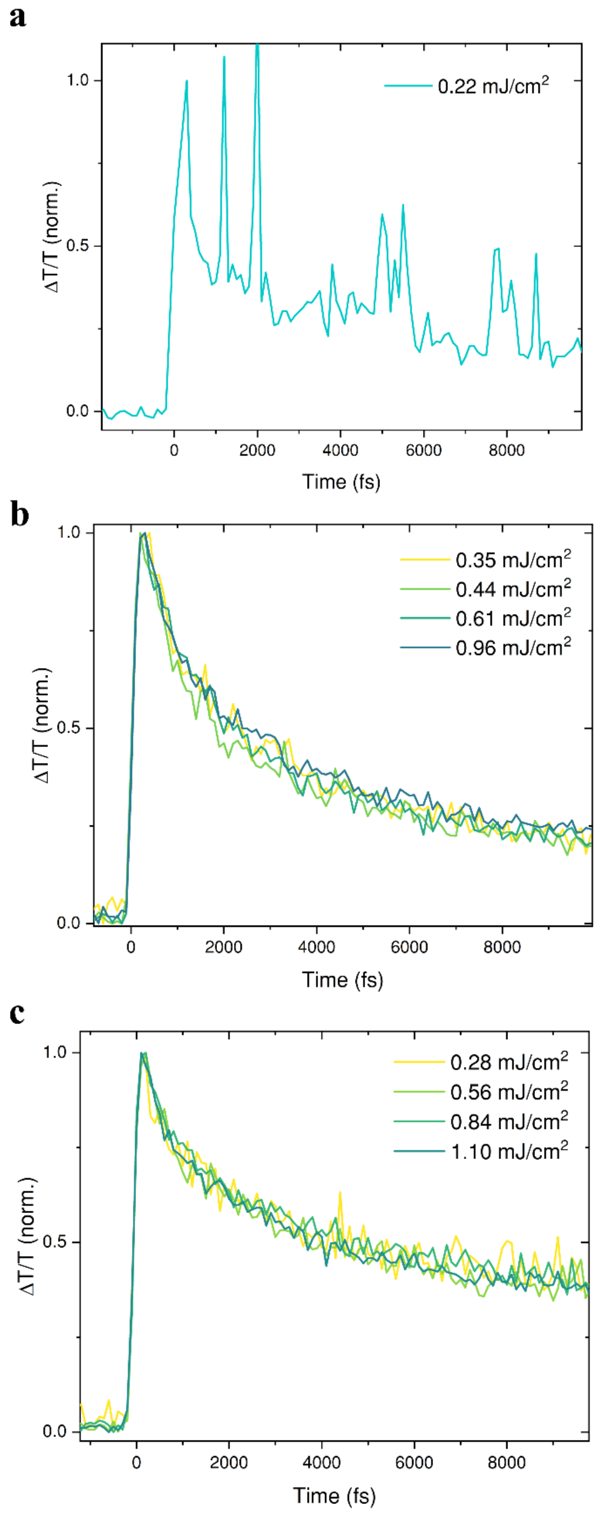

Supplementary Figure S18. Normalized kinetics for $(PEA)_2PbI_4$ (a) and fluence-dependent kinetics for $(Pyr\text{-}C_2)_2PbI_4$ (b) and $(Pyr\text{-}C_4)_2PbI_4$ (c), probed at 2.34 eV (530 nm), 2.58 eV (480 nm) and 2.48 eV (500 nm), respectively.

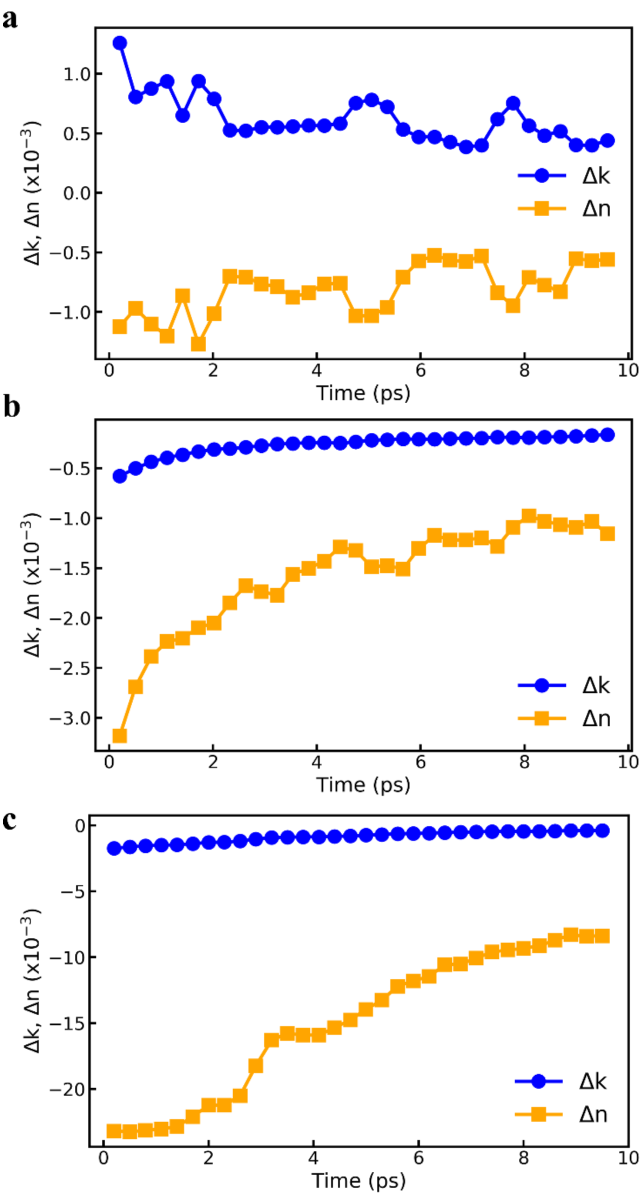

Supplementary Figure S19. Δn (orange) and Δk (dark blue) for $(PEA)_2PbI_4$ (a) $(Pyr\text{-}C_2)_2PbI_4$ (b) and $(Pyr\text{-}C_4)_2PbI_4$ (c) as a result of the TAM fitting. Corresponding Δz and MSD are shown in Figure 4 of the main text.

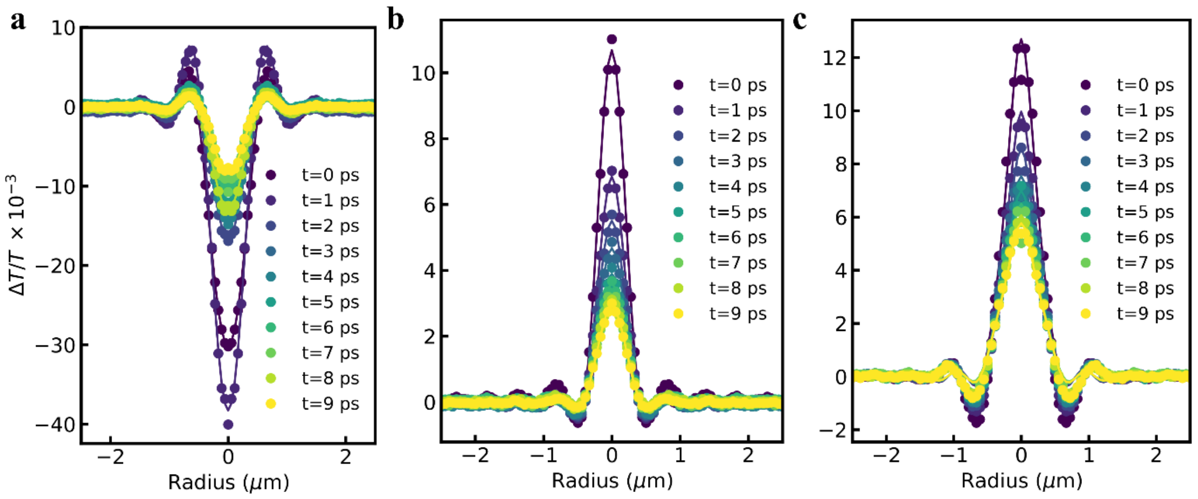

Supplementary Figure S20. Radially averaged ΔT/T images and their corresponding fits for various time points in $(PEA)_2PbI_4$ (a), $(Pyr\text{-}C_2)_2PbI_4$ (b) and $(Pyr\text{-}C_4)_2PbI_4$ (c).

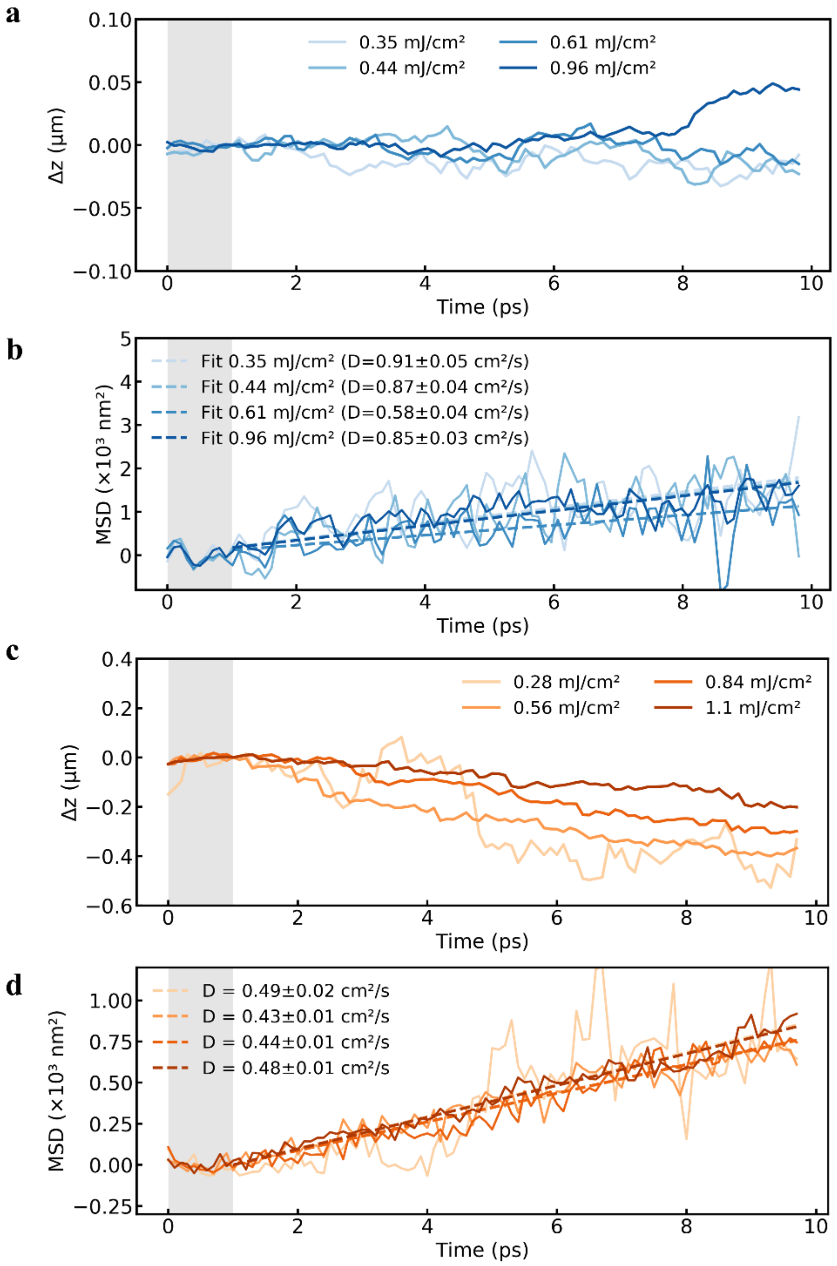

Supplementary Figure S21. Fluence-dependent Δz and MSD for $(Pyr\text{-}C_2)_2PbI_4$ (a, b) and $(Pyr\text{-}C_4)_2PbI_4$ (c,d).

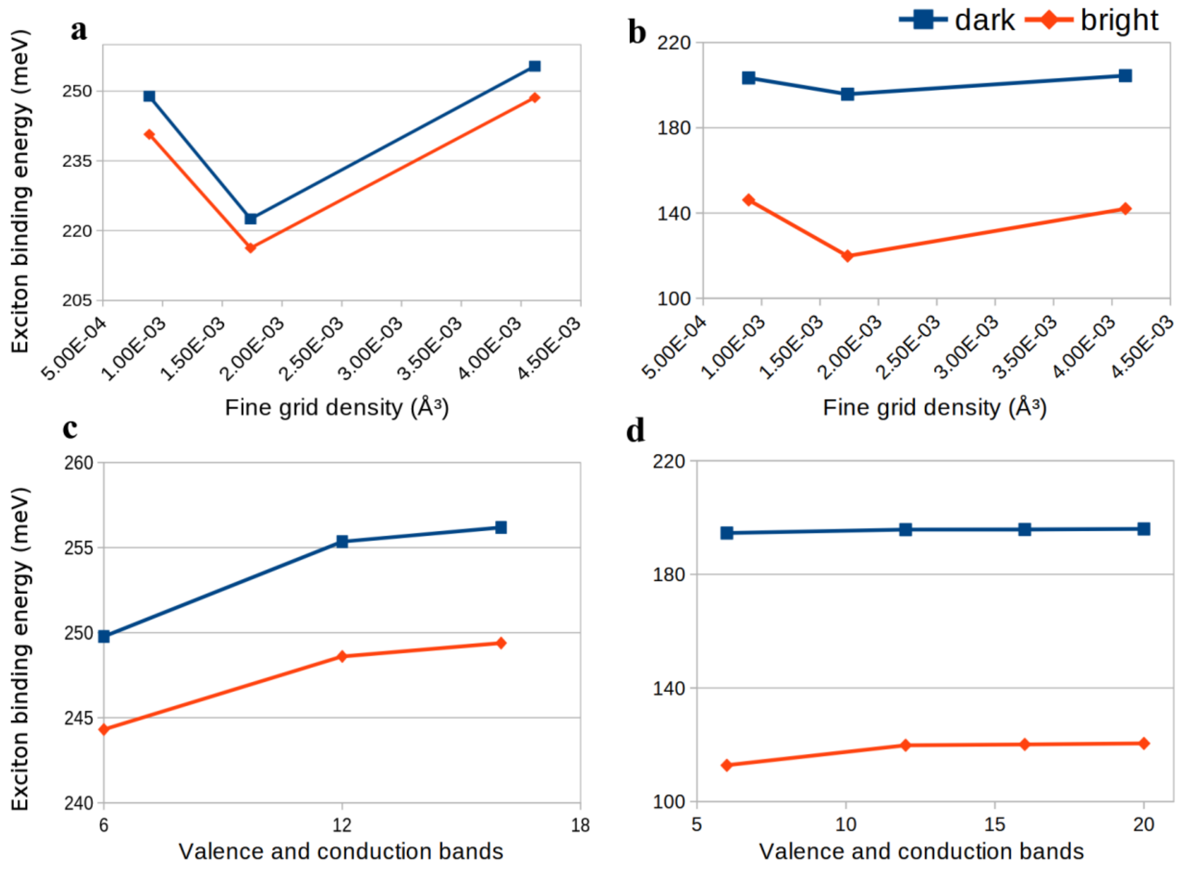

Supplementary Figure S22. Convergence parameters for the exciton binding energies of (Pyr-C_2)$_2$PbI_4 (a and c) and (Pyr-C_4)$_2$PbI_4 (b and d), defined as the difference between the *GW* bandgap and the lowest energy excited state, either the lowest bright (orange) or the lowest dark (blue) state. The convergence of the binding energy with respect to the density of the fine grid used for solving the BSE via interpolation from a 3x3x1 grid (a-b) and the convergence of the binding energy with respect to the number of states around the bandgap included in absorption calculations (c-d).

Supplementary Note 1. $(Pyr\text{-}C_4)_2PbI_4$ exciton fine structure analysis.

In the main manuscript, we discuss the presence of excited states with mixed characteristics of organic-inorganic and inorganic-inorganic transitions in $(Pyr\text{-}C_4)_2PbI_4$, resulting in stronger exciton delocalization. This material also exhibits an OOP state with a much larger absorption coefficient relative to the brightest IP states when compared to the other two 2D perovskites. Here, we highlight in more depth, the fine structure underpinning these OOP features and the mixing of the two types of transitions.

Fig. 3e in the main article shows that the onset of the excitonic absorption spectrum of $(Pyr\text{-}C_4)_2PbI_4$ is much more densely populated with excitonic states below the bandgap than it is in $(PEA)_2PbI_4$ and other similar 2D perovskites. This can be attributed to the increased density of states around the VBM due to the presence of several organic bands in this energy range.

Typically, the onset of a 2D perovskite consists of a series of four excited states, one dark singlet state and three bright triplet states, of which two are IP and one is OOP.[6,7] The origin of these bright triplet states can be attributed to spin-orbit effects in the CBM, which are mostly comprised of Pb 6p orbitals. Spin-orbit effects couple the p_x and p_y orbitals to spin states, providing the bright triplet states with their orthogonal dipole transition moments. The VBM, on the other hand, is comprised of Pb s and, to a lesser extent, I p orbitals, the Bloch states here exhibiting much less anisotropy and no strong spin-orbit effects.

If the Bloch states arising from the organic VBM bands also do not exhibit strong spin-orbit effects, as is typical for light atoms such as carbon, it would not be surprising that similar dark singlet and bright triplet series are observed in $(Pyr\text{-}C_4)_2PbI_4$. Indeed, upon inspection of the oscillator strengths of the excitonic states along the lattice vectors in Supplementary Table 1, we can see that the onset of the fine structure consists of four, instead of one, of such series, leading to a total of sixteen states. Each of these series does not overlap with the previous and follows the same excitonic ordering as is observed in 2D perovskites with type-I band alignment. Namely, a dark singlet, two orthogonally polarized in-plane (IP) bright triplets, followed by an out-of-plane (OOP) bright triplet state.

As the highest inorganic band is positioned at slightly lower energy than the VBM, the contributions from transitions from this band to the excited states increase with higher exciton energies, up to the fourth series of four. The oscillator strengths of the bright states increase linearly with the contribution from inorganic-inorganic transitions to the state. Since a larger fraction of the hole part of the excitonic wavefunction resides in the inorganic layer, the oscillator strength increases linearly with the electron-hole overlap. This results in excitons that combine transitions from two very different valence bands, both chemically and in terms of spatial localization. Due to this BSE-level hybridization of states, these excitons can exhibit properties characteristic of both types of transitions. Namely the brightness of inorganic-inorganic transitions and the OOP and charge-transfer character of organic-inorganic transitions. The latter of these would ordinarily be dark due to the lack of overlap between the electron and hole components of these excited states.

As can be seen in Supplementary Figure 23a-b, within each series of four, the second (X) and third (Y) states are polarized along the [100] and [010] directions (IP) respectively (directions as indicated in Fig. 1a of the main text). With their oscillator strength increasing for each higher energy series, up to the third one (states 10 and 11). Beyond these states, the inorganic

contributions drop back down to almost zero, making higher energy states dark in the IP directions.

Displayed in Fig. S23d is the fourth state (Z) of each series. A similar increase in oscillator strength can be seen for each subsequent series along the OOP axis, [001]. The increase in oscillator strength for the OOP states, however, persists up to the fourth instead of the third series, at the sixteenth state. It is this state which has the highest inorganic contribution out of all the optically bright excitons, and which is responsible for the significantly enhanced OOP brightness relative to the IP states compared to most other 2D perovskites.

Finally, Fig. S23c shows the first state (D) of each series, which is dark. However, these states in the first two series (one and five) are considerably brighter along the [010] direction than in the rest of the series, by two to three orders of magnitude. This is likely also due to an increased contribution from inorganic-inorganic transitions to these two states, with the fifth state possessing the second largest degree of BSE-level hybridization. This state shows a strikingly large anisotropy between the in-plane directions: The oscillator strength of the polarization along the [010] direction is four orders of magnitude larger than that along the [100] direction. We note that the [010] direction is perpendicular to the π-π stacking direction of the aromatic groups.

In short, the excitonic spectrum consists of four series of four excitons. Within each series, the state ordering indicated by their polarization is D>X>Y>Z. Each consecutively higher energy series displays excitons with increasingly large degrees of BSE-level hybridization between the organic and inorganic layers, up to a maximum. For the dark state, this maximum occurs in series 2, for the IP states, in series 3, and for the OOP state in series 4. Higher-energy states beyond these sixteen do not seem to be organised in these series of four and are mostly dark.

Supplementary Table S3: Table listing the sixteen lowest-energy excited states in (Pyr-$C_4)_2PbI_4$, their energies and the components of the dipole transition moment along the three reciprocal lattice vectors.

State \ Pol. direction	Energy (eV)	Oscillator strength [100]	Oscillator strength [010]	Oscillator strength [001]
1	1.7850	0.28E-03	0.62E+01	0.39E-04
2	1.7856	0.64E+02	0.59E-01	0.12E-01
3	1.7862	0.13E+00	0.72E+02	0.71E-03
4	1.7869	0.41E+01	0.42E-02	0.90E+01
5	1.8102	0.84E-02	0.15E+02	0.13E-03
6	1.8165	0.53E+03	0.27E+01	0.36E+00
7	1.8183	0.32E+01	0.47E+03	0.17E-04
8	1.8212	0.55E+01	0.58E-02	0.12E+02
9	1.8246	0.43E-02	0.32E-01	0.13E-03
10	1.8284	0.68E+03	0.79E+01	0.61E+00
11	1.8302	0.15E+02	0.97E+03	0.47E-02
12	1.8532	0.13E+02	0.15E-02	0.69E+02
13	1.8546	0.48E-05	0.14E-03	0.37E-05
14	1.8547	0.12E+02	0.15E+00	0.16E-01
15	1.8547	0.32E+00	0.16E+02	0.12E-03
16	1.8647	0.64E+02	0.15E-01	0.35E+03

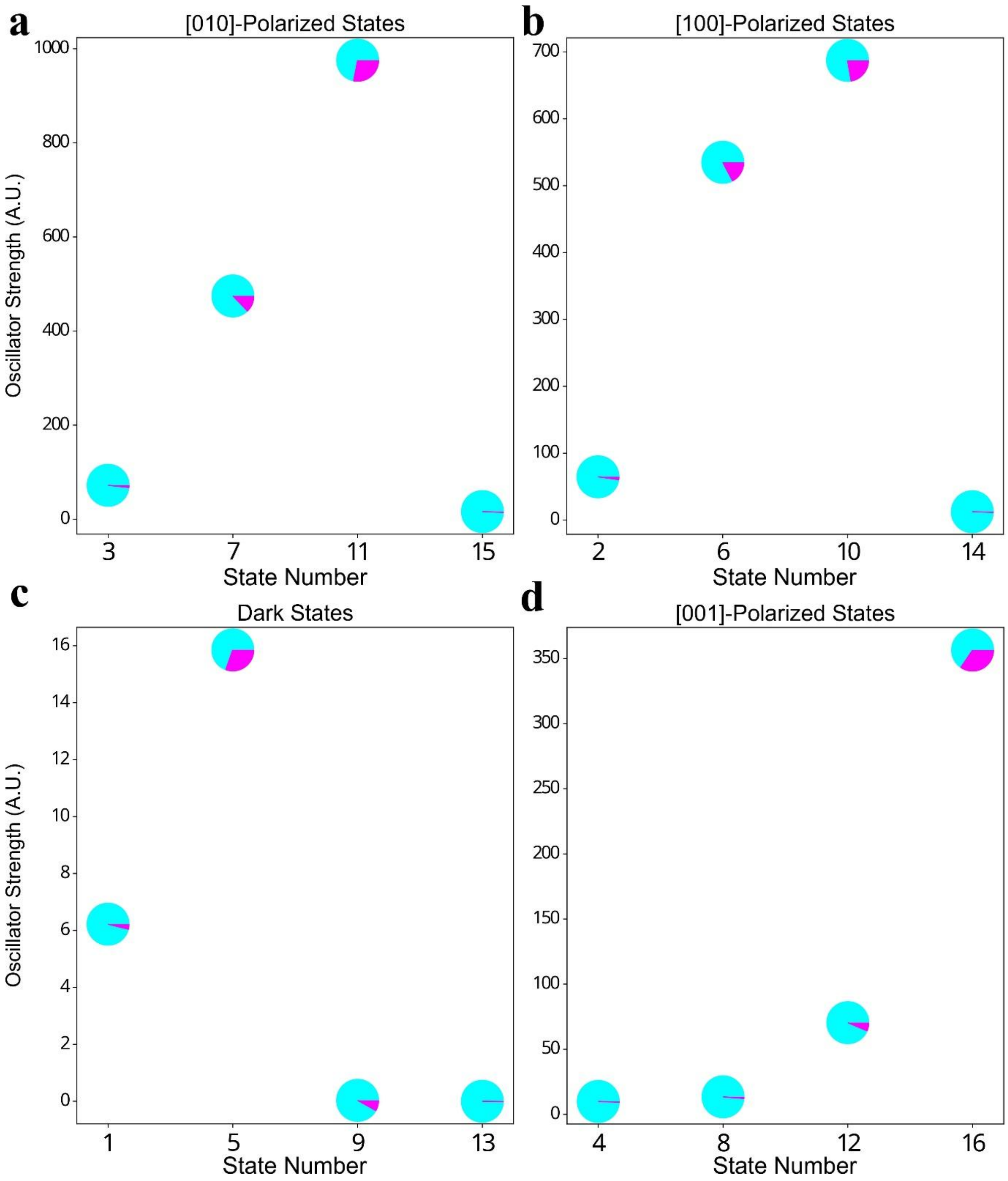

Supplementary Figure S23. The total oscillator strength of the first 16 excited states, numbered in order of increasing energy. The cyan part represents the contribution from organic-inorganic transitions and the magenta the contribution from inorganic-inorganic transitions. The states with their polarization along the in-plane directions (a-b), the dark states (c) and the states with out-of-plane polarization (d) are displayed separately to highlight the trend of increasing oscillator strength with the inorganic-inorganic contribution.

Supplementary Note 2. Transient absorption microscopy model.

We use a modified version of the analytical model by Ashoka et al.[8] to be able to describe 3D diffusion in thick single-crystalline films. We start with the equation to describe the pump intensity I_{pu} as a function of the radius r and crystal depth z. We assume that the pump beam is a TEM_{00} mode allowing us to average out polarisation effects:

$$I_{pu}(r,z) = I_0 \left\{ \frac{\sigma_0}{\sigma(z)}^2 \right\} \exp \left\{ \frac{-r^2}{2\sigma(z)^2} - \frac{\omega_{pu} \alpha z}{c} \right\}$$

The σ and z quantities are of interest to describe in-plane (lateral) and out-of-plane (vertical) diffusion, respectively. The I_{pu} is described as a Gaussian beam attenuated through the depth of the crystal as determined by the pump frequency ω_{pu} and thickness-corrected absorption (i.e. a dimensionless quantity indicating the fraction of absorbed photons) α. This term may be neglected for sufficiently thin films. As our currently studied systems are thick crystals (L ~ 1 µm) with an estimated α of 1 (Fig. S4), we include this term explicitly. Similarly, the z-dependence of σ,

$$\sigma(z) = \sigma_0 \sqrt{1 + (\frac{z}{z_r})^2}$$

can be neglected when the film thickness is much smaller than the Rayleigh range,

$$z_r = \frac{\pi {w_0}^2 \omega_{pu}}{c}$$

where w_0 is the beam waist (i.e. the radial size of the beam at its narrowest point). For a diffraction-limited λ=450 nm beam, ω_{pu} = 6.7x10^{14} Hz, and w_0 ~ 230 nm, we estimate z_r~ 0.4 µm. As the crystal thickness exceeds z_r we include the z-dependence for σ in this work. Hence, both the effects of a decreasing beam waist and the number of photons throughout the crystal depth are taken into account. Making use of the proportionality between $\Delta\tilde{n}$ and I_{pu}

$$\Delta\tilde{n}(r,z) = (\Delta n_0 + i\Delta k_0) I_{pu}(r,z)$$

where $\Delta n_0 + i\Delta k_0$ are the real and imaginary parts of the transient refractive index centred about the imaging plane z_0, we retrieve the complex transient refractive index $\Delta\tilde{n}(r,z)$ by taking into account the effects of a decreasing beam waist and number of photons throughout the crystal depth:

$$\Delta\tilde{n}(r,z) = \frac{1}{N_0}(\Delta n_0 + i\Delta k_0) \frac{1}{1 + (\frac{z}{z_r})^2} \exp \left\{ \frac{-r^2}{2(\sigma_0^2 * (1 + \left(\frac{z}{z_r}\right)^2))} - \frac{\omega_{\mathrm{pu}} \alpha z}{c} \right\}$$

where N_0 is the normalization constant,

$$N_0 = \sqrt{2}\pi^{\frac{3}{2}} \frac{1}{4} \sigma_0 z_r \boldsymbol{K_0}(\frac{\omega_{pu} \alpha z}{c})$$

and $\boldsymbol{K_0}$ is the second kind version of the Struve function. Calculating the near-field electric field of a propagating probe plane wave through the 3D $\Delta\tilde{n}(r, z)$ function should formally be done using the transfer matrix method. Alternatively, the finite-difference time-domain method could be applied to numerically solve this problem. To make our model computationally feasible, however, we assume the same diffraction equation that was used in Ref. 8 and follow the rest of the mathematical procedure used therein. This involves a Fourier-transformation to calculate the far-field electric field at the objective's input aperture and a Richards-Wolf type integral of this far-field electric field to calculate its image on the camera, which considers diffraction of a spherical wave by the circular aperture of the lens as the main source of the point spread function. Note that even though $\Delta\tilde{n}(r, z)$ is a function of both r and z, we only have optical access to r in our microscopy setup and therefore we fit our data to this function by explicitly considering r as a variable, but z as a parameter. Then, with pre-knowledge of $\Delta n_0 + i\Delta k_0$ from our Kramers-Kronig analysis (Figure S17) and estimations of z_r and α, the radially averaged *ΔT/T*(r) images are fitted for each time point to retrieve our time-varying in-plane (IP) and out-of-plane (OOP) diffusion parameters of interest: σ_0 (t) and z (t), respectively. To suppress artificial high-frequency oscillations arising from noise and overfitting, and to ensure smooth parameter evolution, we applied temporal smoothing to the radial profile prior to fitting and imposed dynamically constrained bounds on the fit parameters.

When OOP diffusion is negligible, our model is able to provide IP diffusion constants through retrieval of the time-dependent σ. However, for sufficiently fast OOP diffusion on the timescales of IP diffusion, the two become coupled, resulting in an underestimation of the absolute IP diffusion constant. This may be understood by considering that the sides of the exciton carrier distribution experience a larger z-gradient due to the Rayleigh range of the pump beam. Therefore, due to the loss in IP carrier gradient associated with strong OOP transport, the effective MSD is expected to be suppressed, as has been observed for $(Pyr\text{-}C_4)_2PbI_4$ (Fig. 4d, main text).

Another complication is that photoexcitation of $(Pyr\text{-}C_4)_2PbI_4$ should lead to a combination of interlayer excitons involving the pyrene orbitals, as well as the intralayer excitons involving the PbI_4^{2-} bands only (Supplementary Note 1). The diffusion of two coupled exciton populations is non-linear and may result in an apparent contraction, as has been observed for trapped and non-trapped excitons in 2D perovskites,[9–11] bright and lower-lying dark excitons in transition metal dichalcogenides,[12] and finally, dark OOP and bright IP excitons in a band type-II 2D perovskite.[13] Due to the fast excitonic decay in $(Pyr\text{-}C_4)_2PbI_4$, we are unable to access the longer timescales required to probe the potential interconversion between these exciton populations.[13]

A final remark is that due to the rapid degradation of the less stable $(PEA)_2PbI_4$, we were unable to measure multiple fluences for this material. The $(Pyr\text{-}C_2)_2PbI_4$ and $(Pyr\text{-}C_4)_2PbI_4$ samples are markedly more photo-stable than the $(PEA)_2PbI_4$ samples. An enhanced photo-stability of 2D perovskites containing an extended conjugated organic cation as compared to PEA has been previously reported in the literature.[14]

References

(1) Van Gompel, W. T. M.; Herckens, R.; Van Hecke, K.; Ruttens, B.; D'Haen, J.; Lutsen, L.; Vanderzande, D. Low-Dimensional Hybrid Perovskites Containing an Organic Cation with an Extended Conjugated System: Tuning the Excitonic Absorption Features. *Chem. Nano Mat.* **2019**, *5* (3), 323–327. DOI:10.1002/cnma.201800561.

(2) *TURBOMOLE V7.6 2021, a development of University of Karlsruhe and Forschungszentrum Karlsruhe GmbH, 1989-2007*. TURBOMOLE GmbH, since 2007; available from https://www.turbomole.org.

(3) Balasubramani, S. G.; Chen, G. P.; Coriani, S.; Diedenhofen, M.; Frank, M. S.; Franzke, Y. J.; Furche, F.; Grotjahn, R.; Harding, M. E.; Hättig, C.; Hellweg, A.; Helmich-Paris, B.; Holzer, C.; Huniar, U.; Kaupp, M.; Marefat Khah, A.; Karbalaei Khani, S.; Müller, T.; Mack, F.; Nguyen, B. D.; Parker, S. M.; Perlt, E.; Rappoport, D.; Reiter, K.; Roy, S.; Rückert, M.; Schmitz, G.; Sierka, M.; Tapavicza, E.; Tew, D. P.; Van Wüllen, C.; Voora, V. K.; Weigend, F.; Wodyński, A.; Yu, J. M. TURBOMOLE: Modular Program Suite for Ab Initio Quantum-Chemical and Condensed-Matter Simulations. *J. Chem. Phys.* **2020**, *152* (18), 184107. DOI:10.1063/5.0004635.

(4) Xue, J.; Wang, R.; Chen, X.; Yao, C.; Jin, X.; Wang, K. L.; Huang, W.; Huang, T.; Zhao, Y.; Zhai, Y.; Meng, D.; Tan, S.; Liu, R.; Wang, Z. K.; Zhu, C.; Zhu, K.; Beard, M. C.; Yan, Y.; Yang, Y. Reconfiguring the Band-Edge States of Photovoltaic Perovskites by Conjugated Organic Cations. *Science* **2021**, *371* (6529), 636–640. DOI:10.1126/science.abd4860.

(5) Jingjing Xue. *Internal communication*.

(6) Becker, M. A.; Vaxenburg, R.; Nedelcu, G.; Sercel, P. C.; Shabaev, A.; Mehl, M. J.; Michopoulos, J. G.; Lambrakos, S. G.; Bernstein, N.; Lyons, J. L.; Stöferle, T.; Mahrt, R. F.; Kovalenko, M. V.; Norris, D. J.; Rainò, G.; Efros, A. L. Bright Triplet Excitons in Caesium Lead Halide Perovskites. *Nature* **2018**, *553* (7687), 189–193. DOI:10.1038/nature25147.

(7) Even, J.; Pedesseau, L.; Dupertuis, M.-A.; Jancu, J.-M.; Katan, C. Electronic Model for Self-Assembled Hybrid Organic/Perovskite Semiconductors: Reverse Band Edge Electronic States Ordering and Spin-Orbit Coupling. *Phys. Rev. B* **2012**, *86*, 205301. DOI:10.1103/PhysRevB.86.205301.

(8) Ashoka, A.; Gauriot, N.; Girija, A. V.; Sawhney, N.; Sneyd, A. J.; Watanabe, K.; Taniguchi, T.; Sung, J.; Schnedermann, C.; Rao, A. Direct Observation of Ultrafast Singlet Exciton Fission in Three Dimensions. *Nat. Commun.* **2022**, *13* (1), 1–8. DOI:10.1038/s41467-022-33647-5.

(9) Ziegler, J. D.; Zipfel, J.; Meisinger, B.; Menahem, M.; Zhu, X.; Taniguchi, T.; Watanabe, K.; Yaffe, O.; Egger, D. A.; Chernikov, A. Fast and Anomalous Exciton Diffusion in Two-Dimensional Hybrid Perovskites. *Nano Lett.* **2020**, *20* (9), 6674–6681. DOI:10.1021/ACS.NANOLETT.0C02472.

(10) Seitz, M.; Meléndez, M.; Alcázar-Cano, N.; Congreve, D. N.; Delgado-Buscalioni, R.; Prins, F.; Seitz, M.; Prins, F.; Congreve, D. N.; Meléndez, M.; Alcázar-Cano, N.; Delgado-Buscalioni, R. Mapping the Trap-State Landscape in 2D Metal-Halide Perovskites Using

Transient Photoluminescence Microscopy. *Adv. Opt. Mater.* **2021**, *9* (18), 2001875. DOI:10.1002/ADOM.202001875.

(11) Kurilovich, A. A.; Mantsevich, V. N.; Chechkin, A. V.; Palyulin, V. V. Negative Diffusion of Excitons in Quasi-Two-Dimensional Systems. *Phys. Chem. Chem. Phys.* **2024**, *26* (2), 922–935. DOI:10.1039/D3CP03521B.

(12) Rosati, R.; Perea-Causín, R.; Brem, S.; Malic, E. Negative Effective Excitonic Diffusion in Monolayer Transition Metal Dichalcogenides. *Nanoscale* **2019**, *12* (1), 356–363. DOI:10.1039/C9NR07056G.

(13) Ou, Z.; Wang, C.; Tao, Z.-G.; Li, Y.; Li, Z.; Zeng, Y.; Li, Y.; Shi, E.; Chu, W.; Wang, T.; Xu, H. Organic Ligand Engineering for Tailoring Electron–Phonon Coupling in 2D Hybrid Perovskites. *Nano Lett.* **2024**. DOI:10.1021/ACS.NANOLETT.4C00463.

(14) Park, I.-H.; Chu, L.; Leng, K.; Fong Choy, Y.; Liu, W.; Abdelwahab, I.; Zhu, Z.; Ma, Z.; Chen, W.; Xu, Q.-H.; Eda, G.; Ping Loh, K.; Park, I.; Chu, L.; Leng, K.; Choy, Y. F.; Liu, W.; Abdelwahab, I.; Zhu, Z.; Ma, Z.; Chen, W.; Xu, Q.; Eda, G.; Loh, K. P. Highly Stable Two-Dimensional Tin(II) Iodide Hybrid Organic–Inorganic Perovskite Based on Stilbene Derivative. *Adv. Funct. Mater.* **2019**, *29* (39), 1904810. DOI:10.1002/ADFM.201904810.
